# Circular RNA circMYLK4 shifts energy metabolism from glycolysis to OXPHOS by binding to the calcium channel auxiliary subunit CACNA2D2

**DOI:** 10.1016/j.jbc.2024.107426

**Published:** 2024-05-30

**Authors:** Haigang Cao, Chenchen Li, Xiaohui Sun, Jinjin Yang, Xiao Li, Gongshe Yang, Jianjun Jin, Xine Shi

**Affiliations:** Laboratory of Animal Fat Deposition and Muscle Development, Key Laboratory of Animal Genetics, Breeding and Reproduction of Shaanxi Province, College of Animal Science and Technology, Northwest A&F University, Yangling, Shaanxi, China

**Keywords:** skeletal muscle, energy metabolism, glycolysis, fatty acid oxidation, calcium, circRNA

## Abstract

Skeletal muscle is heterogeneous tissue, composed of fast-twitch fibers primarily relying on glycolysis and slow-twitch fibers primarily relying on oxidative phosphorylation. The relative expression and balance of glycolysis and oxidative phosphorylation in skeletal muscle are crucial for muscle growth and skeletal muscle metabolism. Here, we employed multi-omics approaches including transcriptomics, proteomics, phosphoproteomics, and metabolomics to unravel the role of circMYLK4, a differentially expressed circRNA in fast and slow-twitch muscle fibers, in muscle fiber metabolism. We discovered that circMYLK4 inhibits glycolysis and promotes mitochondrial oxidative phosphorylation. Mechanistically, circMYLK4 interacts with the voltage-gated calcium channel auxiliary subunit CACNA2D2, leading to the inhibition of Ca^2+^ release from the sarcoplasmic reticulum. The decrease in cytoplasmic Ca^2+^ concentration inhibits the expression of key enzymes, PHKB and PHKG1, involved in glycogen breakdown, thereby suppressing glycolysis. On the other hand, the increased fatty acid β-oxidation enhances the tricarboxylic acid cycle and mitochondrial oxidative phosphorylation. In general, circMYLK4 plays an indispensable role in maintaining the metabolic homeostasis of skeletal muscle.

Mammalian skeletal muscle is a highly heterogeneous tissue composed of multiple types of fibers. However, these fibers are not fixed units but highly adaptable entities capable of altering their phenotypic characteristics in response to changing functional demands and various signals (1). The flexibility of skeletal muscle in utilizing carbohydrates or lipids is associated with the expression of different structural proteins, which ultimately define distinct contraction characteristics (2). In small mammals, slow muscles such as the soleus are predominantly composed of slow oxidative myofibers expressing slow type 1 myosin heavy chain (MyHC) and fast oxidative myofibers expressing type 2A or type 2X MyHC. Type 1, type 2A, and type 2X muscle fibers are considered to be oxidative muscle fibers that use lipids extensively to meet most of their energy requirements under aerobic conditions in mice. In contrast, fast muscles, such as extensor digitorum longus, gastrocnemius, and tibialis anterior, are mainly composed of fast glycolytic muscle fibers, express MyHC predominance type 2B, and use glucose to generate most of their energy anaerobically (3).

Although both glycolysis and oxidative phosphorylation (OXPHOS) serve as major cellular pathways for ATP production, glycolysis tends to be kinetically favorable, whereas OXPHOS produces higher ATP (4, 5). The relative balance between OXPHOS and glycolysis varies depending on the tissue. For instance, while cardiac tissue is rich in mitochondria and highly oxidative in its metabolism, proliferating cells in the thymus exhibit a high degree of glycolysis (5). The balance between these two programs may vary during cellular differentiation and in response to environmental stimuli. Activation of immune cells is often accompanied by rewiring toward glycolysis, while differentiation of stem cells can lead to an increase in OXPHOS (6, 7). Oxidative fiber types, which primarily rely on OXPHOS, exhibit greater resistance to fatigue compared to glycolytic fiber types (2). It is worth noting that the differential reliance on oxidative phosphorylation and glycolysis can be exploited to gain therapeutic benefits (8, 9).

Circular RNA (circRNA) is a covalently closed RNA molecule formed through a process of back-splicing from precursor mRNA, lacking the 5′ cap and 3′ tail structures (10). Due to their unique structure, circRNAs can evade degradation by exonucleases and exhibit longer half-lives compared to their parental mRNAs (11). Based on this conservative nature, numerous studies have focused on the potential role of circRNAs as promising disease biomarkers (12). Currently, research on circRNAs in the context of metabolism is predominantly focused on diseases. circRNAs can play significant roles in glycolysis by regulating transcription factors (13, 14, 15, 16), signaling pathways (17, 18), transport proteins (19), and enzymes (20, 21, 22). In addition, circRNAs also play important roles in lipid metabolism (23, 24, 25), amino acid metabolism (26), and oxidative respiration (27, 28). However, there is limited knowledge regarding the involvement of circRNAs in skeletal muscle metabolism.

In our previous study, we performed circRNA sequencing on glycolytic and oxidative fiber types in pigs and identified circMYLK4 as a key regulatory factor in fast-to-slow muscle fiber conversion (29). However, its specific function and underlying mechanism require further investigation. In this study, we employed transcriptomic, proteomic, phosphoproteomic, and metabolomic analyses to elucidate the role of circMYLK4. Our results demonstrate that circMYLK4 inhibits the glycolytic process, promotes fatty acid oxidation, and enhances mitochondrial oxidative phosphorylation. Through polysome profiling, RNA pull-down, and RNA immunoprecipitation experiments, we found that circMYLK4 exerts its function by interacting with CACNA2D2 to reduce cytoplasmic calcium ion levels, leads to the inhibition of glycogen breakdown and affects metabolic processes. Overall, circMYLK4 emerges as a crucial regulatory factor in muscle fiber conversion and metabolic processes.

## Results

### circMYLK4 promotes the conversion of fast-twitch fibers to slow-twitch fibers in skeletal muscle

**Figure 1:**
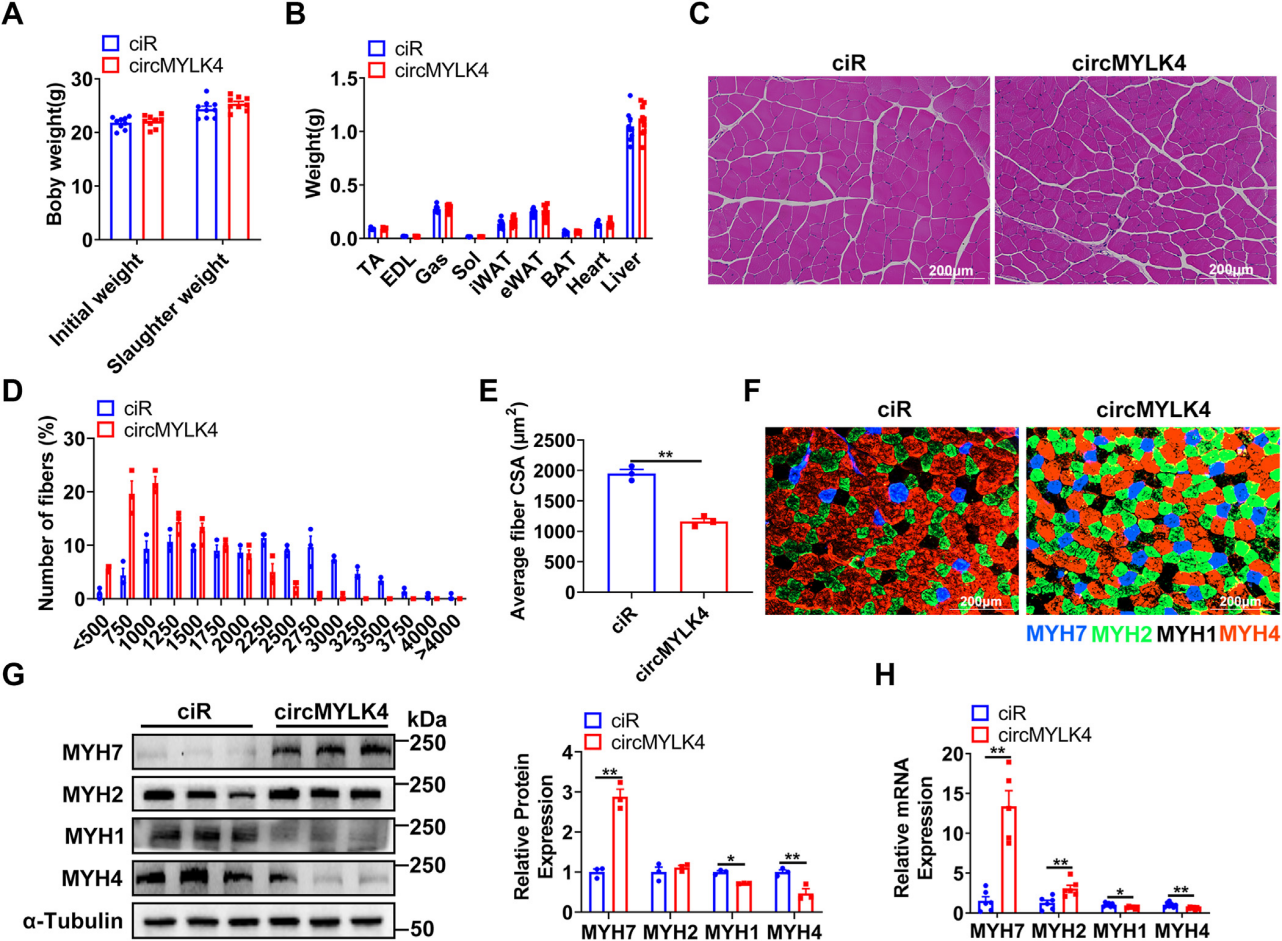
**Overexpression of circMYLK4 leads to a shift in muscle fiber type towards oxidative fibers.***A*, the initial body weight and slaughter weight of ciR and circMYLK4 mice. *B*, the weights of different tissues in ciR and circMYLK4 mice. *C*, H&E staining images of the cross-section of GAS muscle in ciR and circMYLK4 mice. *D* and *E*, distribution of myofiber size and average myofiber cross-sectional area (CSA) of ciR and circMYLK4 GAS muscle. *F*, immunostaining of Type I, IIA, and IIB myofibers in GAS muscle of ciR and circMYLK4 mice. *G*, analysis of protein markers for skeletal muscle fiber types in ciR and circMYLK4 mice using Western blotting. *H*, the mRNA levels of skeletal muscle fiber type markers in ciR and circMYLK4 mice. Data are mean ± SEM of three independent experiments (∗*p* < 0.05, ∗∗*p* < 0.01).

In our previous study, we performed circRNA sequencing on glycolytic and oxidative fibers of pigs, identified, and preliminarily verified that circMYLK4 can promote the expression of oxidative fiber-related genes. We found from the circAtlas 3.0 database that the circular RNA mmu-Mylk4_0004 generated from the mouse MYLK4 gene and the circular RNA hsa-MYLK4_0016 generated from the human MYLK4 gene, both exhibit high sequence similarity with circMYLK4 (Fig. S1, *A* and *B*). This indicates that circMYLK4 is highly conserved in pigs, mice, and humans. Here, we intend to further elucidate the functional role of circMYLK4 by separately administering circMYLK4-AAV and ciR-AAV vectors into the skeletal muscle of mice. However, we found that overexpression of circMYLK4 did not significantly affect body weight, skeletal muscle mass, or other tissue weight in mice (Fig. 1, *A* and *B*). However, H&E staining results showed that overexpression of circMYLK4 led to a leftward shift in the distribution of myofiber sizes and a significant decrease in average muscle fiber cross-sectional area (Fig. 1, *C*–*E*). Further, we assessed myofiber composition in the gastrocnemius muscle based on immunofluorescent staining of myosin heavy chain isoforms. The result showed that overexpression of circMYLK4 significantly increased the proportion of oxidative myofibers (MYH7) and decreased the proportion of glycolytic myofibers (MYH4) (Fig. 1*F*), suggesting that circMYLK4 could promote the formation of oxidized muscle fibers. To confirm the effect of circMYLK4 on muscle fiber types, we examined the expression of muscle fiber subtype genes. RT-qPCR and Western blot results showed that circMYLK4 significantly promoted the slow muscle marker gene MYH7 and significantly inhibited the expression of the fast muscle marker gene MYH4 in mRNA and protein level (Fig. 1, *G* and *H*). This is consistent with previous findings in pigs. Collectively, these findings suggest that circMYLK4 converts glycolytic myofibers into oxidative myofibers.

### Comprehensive characterization of the transcriptome, proteome, and phosphoproteome of circMYLK4-AAV injected into porcine skeletal muscle

**Figure 2:**
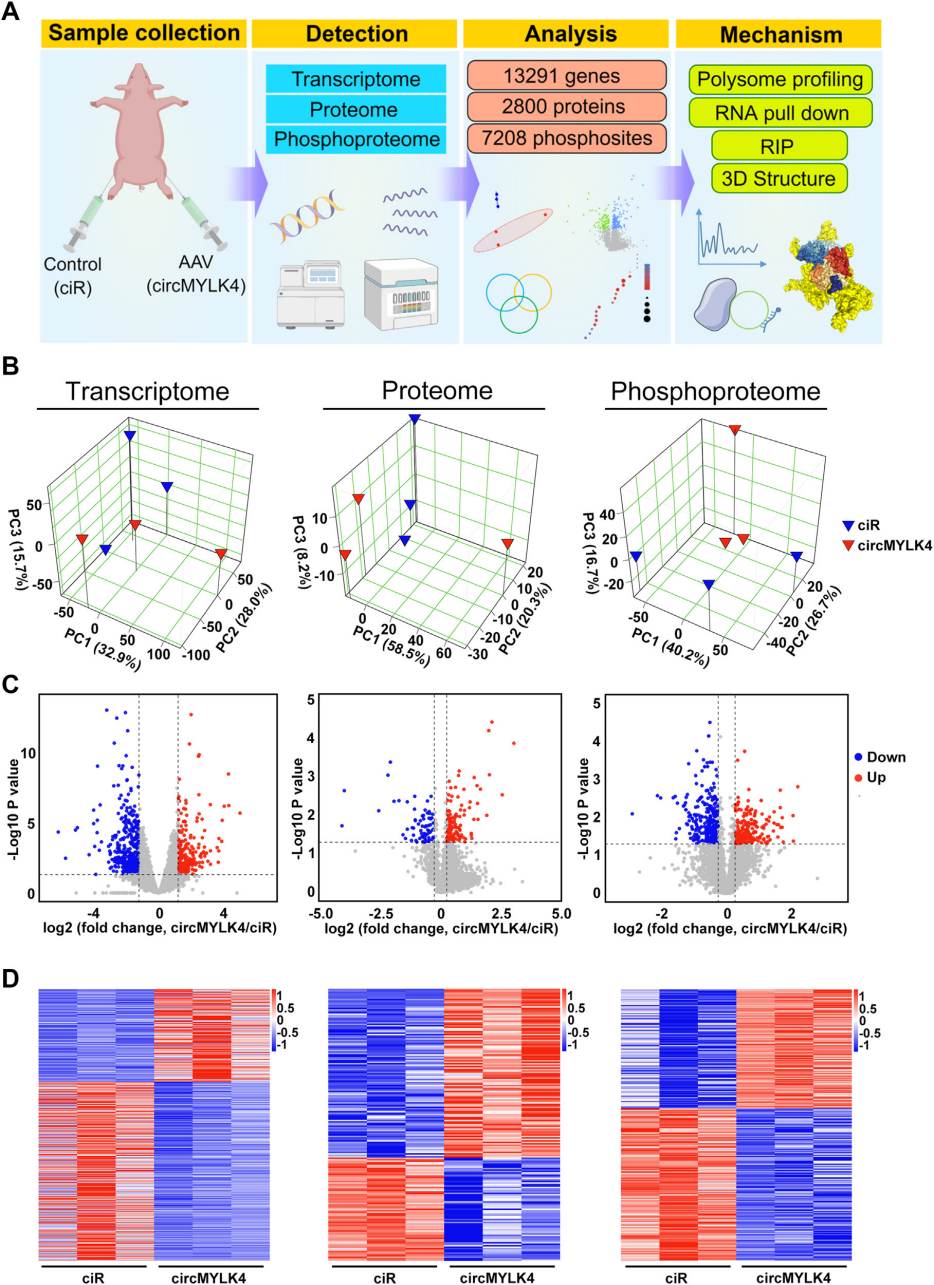
**Multi-omics analysis of skeletal muscle in ciR and circMYLK4 pigs.***A*, the flowchart illustrates the experimental procedure and workflow, using Figdraw drawing. *B*, principal component analysis (PCA) of transcriptome, proteome, and phosphoproteome data. *C*, volcano plots depicting the mRNA, protein, and phosphorylated protein changes between ciR and circMYLK4 skeletal muscles. *Red color* represents significant upregulation, while *blue color* represents significant downregulation. *D*, heatmaps depicting the differentially expressed features in the transcriptome, proteome, and phosphoproteome of ciR and circMYLK4 skeletal muscles. The color bar on the *right* side of the heatmap represents gene expression levels (*blue*, downregulation and *red*, upregulation).

To further clarify the effect of circMYLK4 on skeletal muscle function, we performed a comprehensive analysis of transcriptomic, proteomic, and phosphoproteomic data as described in Experimental procedures (Fig. 2*A*). Principal component analysis (PCA) of our transcriptome, proteome, and phosphoproteome data showed better clustering of replicate samples, representing better quantitative reproducibility (Fig. 2*B*). Overall, we identified 13,291 genes, 2800 proteins, and 7208 phosphosites, of which 949 genes, 210 proteins, and 792 phosphosites were differentially expressed (Fig. 2*C*). The similarity and heterogeneity of samples from different groups in terms of transcriptome, proteome, and phosphoproteome were shown in the heatmaps (Fig. 2*D*).

Furthermore, we performed targeted quantitative validation using Parallel Reaction Monitoring (PRM) to confirm the results of the proteome and phosphoproteome analyses. For the proteome, we selected 21 proteins (Fig. S2*A*) for verification, and the results showed that PRM was completely consistent with the quantitative proteomics results of TMT (Fig. S2*B*). As for the phosphorylation protein group, we selected 20 phosphorylation sites (Fig. S2*C*) for verification, and the results showed that PRM and TMT quantitative phosphorylation modification protein group results were highly consistent (Fig. S2*D*).

### Multi-omics joint analysis of biological processes involved in circMYLK4

**Figure 3:**
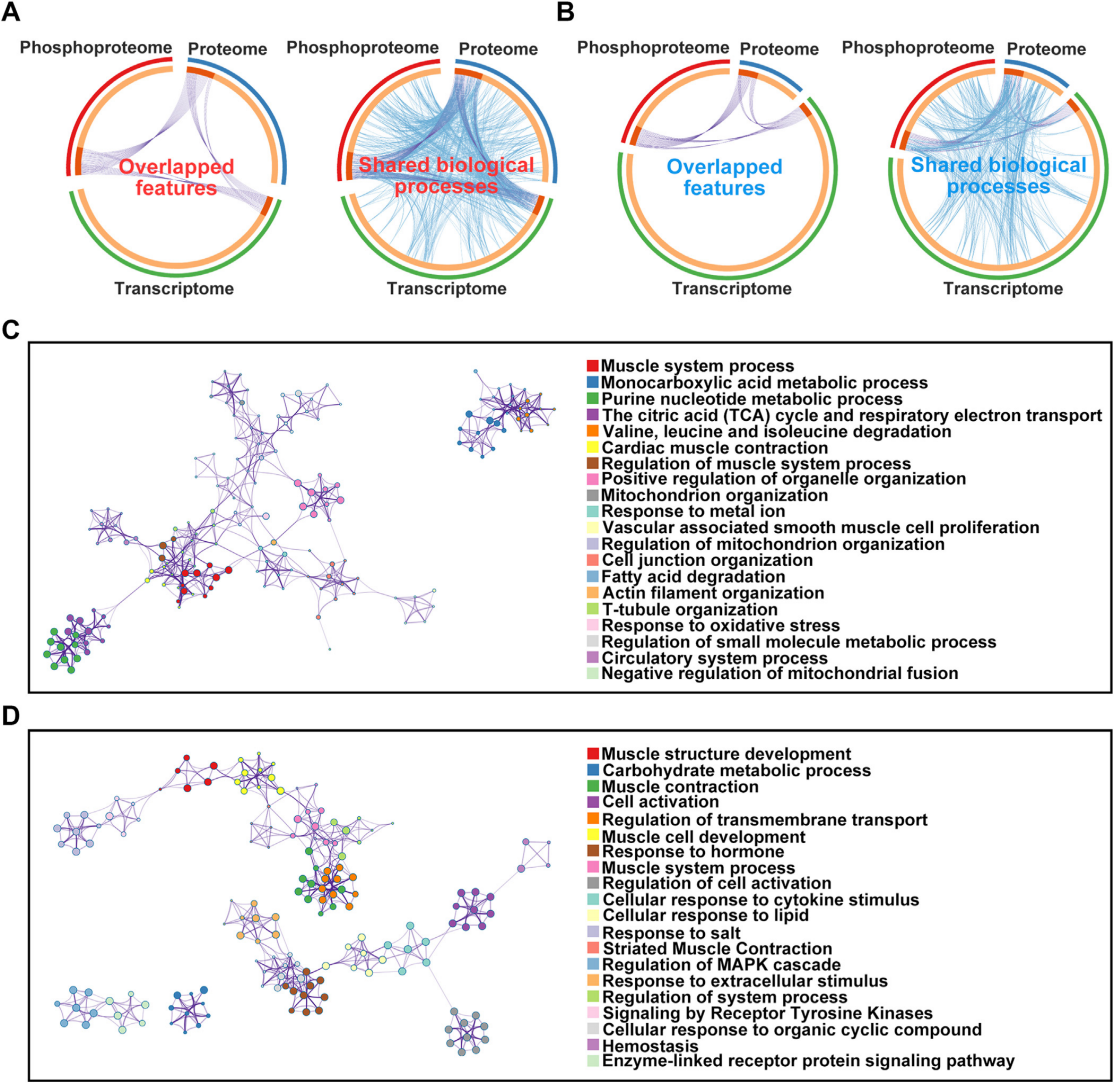
**Visualization of meta-analysis results based on features from transcriptome, proteome, and phosphoproteome data.***A*, the Circos plot illustrates the overlapping genes between the three omics datasets and the overlapping biological processes based on gene functions (upregulated genes in the three omics datasets). The *inner circle* represents the gene lists, with genes shared by multiple lists displayed in deep *orange*, and genes specific to each list displayed in *light orange*. *B*, the Circos plot visualizes the overlapping genes and functions across the three omics datasets (genes that are downregulated in the three omics datasets). *Purple lines* connect the genes shared by the three omics datasets, while *blue lines* connect genes associated with the same biological processes. Network visualization of the enriched biological processes for the upregulated genes (*C*) in the three omics datasets and the downregulated genes (*D*) in the three omics datasets. Each node represents an enriched biological process.

We next explored the biological processes in which circMYLK4 is involved through different combinatorial analyses. Circos (30) was first used to reveal the correlation of features or their annotations between different omics datasets. As shown in Figure 3, *A* and *B*, no matter whether it is up-regulated or down-regulated features, only a few features directly overlap in the three omics datasets, while their functional annotations show extensive overlap, especially for up-regulated features. To pinpoint common pathways involving features that are consistently up- or down-regulated, interaction enrichment analysis was applied. Connected network components of up-regulated features were identified in Figure 3*C*, including the monocarboxylic acid metabolic process, the citric acid (TCA) cycle and respiratory electron transport, mitochondrion organization, fatty acid degradation, *and so on.* For downregulated features, the involved network included carbohydrate metabolic process, and regulation of transmembrane transport (Fig. 3*D*).

**Figure 4:**
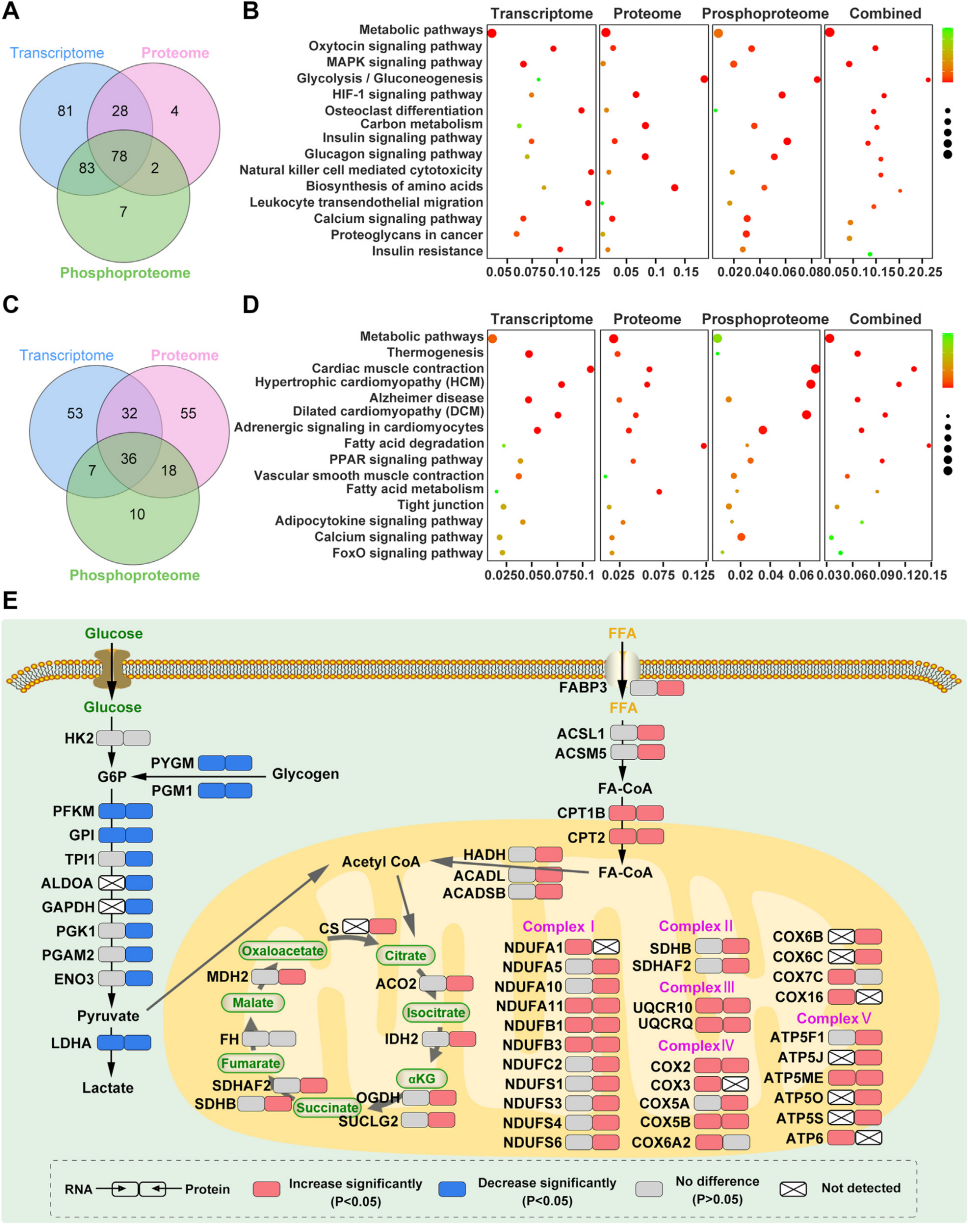
**Multi-omics analysis reveals the impact of circMYLK4 on skeletal muscle pathways.***A*, the Venn diagram illustrates the intersection of enriched pathways among the downregulated genes in the three omics datasets. *B*, the bubble plot displays the top 15 enriched pathways shared by the downregulated genes in the three omics datasets. The color represents the adjusted *p-value*, and the size of the bubbles corresponds to the number of genes within the top 15 enriched pathways. *C*, the Venn diagram illustrates the intersection of enriched pathways among the upregulated genes in the three omics datasets. *D*, the bubble plot displays the top 15 enriched pathways shared by the upregulated genes in the three omics datasets. The color represents the adjusted *p-value*, and the size of the bubbles corresponds to the number of genes within the top 15 enriched pathways. *E*, reprogrammed metabolic pathways in skeletal muscle overexpressing circMYLK4. Protein and mRNA changes in skeletal muscle overexpressing circMYLK4 compared to the control group.

In order to clarify which signaling pathways circMYLK4 is involved in, we performed pathway enrichment analysis on differentially up-regulated and differentially down-regulated features in the three omics. The results showed that 270 pathways were enriched in the transcriptome, 112 pathways were enriched in the proteome, and 170 pathways were enriched in the phosphoproteome in the downregulated features. We found 78 common pathways (Fig. 4*A*), which were mainly related to metabolism (such as Glycolysis/Gluconeogenesis, Carbon metabolism, Insulin signaling pathway, and biosynthesis of amino acids) (Fig. 4*B*). For the up-regulated signature, 128 pathways were enriched to the transcriptome, 141 pathways were enriched to the proteome, and 71 pathways were enriched to the phosphoproteome, among which 36 pathways were common (Fig. 4*C*). These pathways are also mainly related to metabolism (such as Thermogenesis and Fatty acid metabolism) (Fig. 4*D*). These results suggest that circMYLK4 may be involved in skeletal muscle energy metabolism.

To investigate the systemic changes in metabolic pathways with greater precision, we subsequently conducted in-depth analyses of transcriptomic and proteomic data. The downregulation of glycolysis (PFKM, GPI, TPI, PGK1, PGAM2, and ENO3) and upregulation of fatty acid oxidation (FABP3, ACSL1, CPT1B, CPT2, and ACADL), TCA cycle (ACO2, IDH2, OGDH, SDHB, and MDH2), OXPHOS (NDUFA11, NDUFB1, NDUFB3, UQCRC10, UQCRQ, COX2, COX5B, and ATP5ME) components that we observed at mRNA and/or protein levels indicated circMYLK4 alters skeletal muscle metabolism (Fig. 4*E*). Furthermore, we found that the downregulation of glycolysis was not due to reduced glucose utilization (HK2 was unchanged), but rather to reduced breakdown of muscle glycogen (both mRNA and protein levels of PYGM and PGM1 were significantly downregulated) (Fig. 4*E*). We further examined the glycogen content in skeletal muscle and found that the glycogen content of the circMYLK4 overexpression group was relatively higher. (Fig. S6*A*).

Alterations in skeletal muscle energy metabolism by circMYLK4 prompted us to analyze the abundance of metabolic intermediates central to glycolysis and the TCA cycle. We performed targeted quantification of 68 metabolites related to energy metabolism using liquid chromatography-mass spectrometry (LC-MS). As expected, we observed a significant decrease in the glycolytic intermediate metabolites glucose-6-phosphate and fructose-6-phosphate, as well as the muscle glycogen catabolite glucose-1-phosphate (Fig. S3*A*). In addition, TCA cycle metabolites fumarate, malate, and oxaloacetate were significantly increased, and surprisingly, α-ketoglutarate was significantly decreased (Fig. S3*B*). Glutamine is an important fuel that can contribute glutamate to the TCA cycle (31). Intracellular glutamate may either be converted into α-ketoglutarate to serve as an anaplerotic input into the TCA cycle. We speculate that the decrease in α-ketoglutarate may be due to decreased synthesis of glutamine and glutamate (Fig. S4). In addition, the expression levels of leucine and serine also decreased, which was consistent with the analysis results in Figure 2*C*. While the expression of alanine, tyrosine, and citrulline increased to a certain extent, the expression levels of other amino acids did not change much (Fig. S4).

### circMYLK4 alters the metabolism of muscle satellite cells

**Figure 5:**
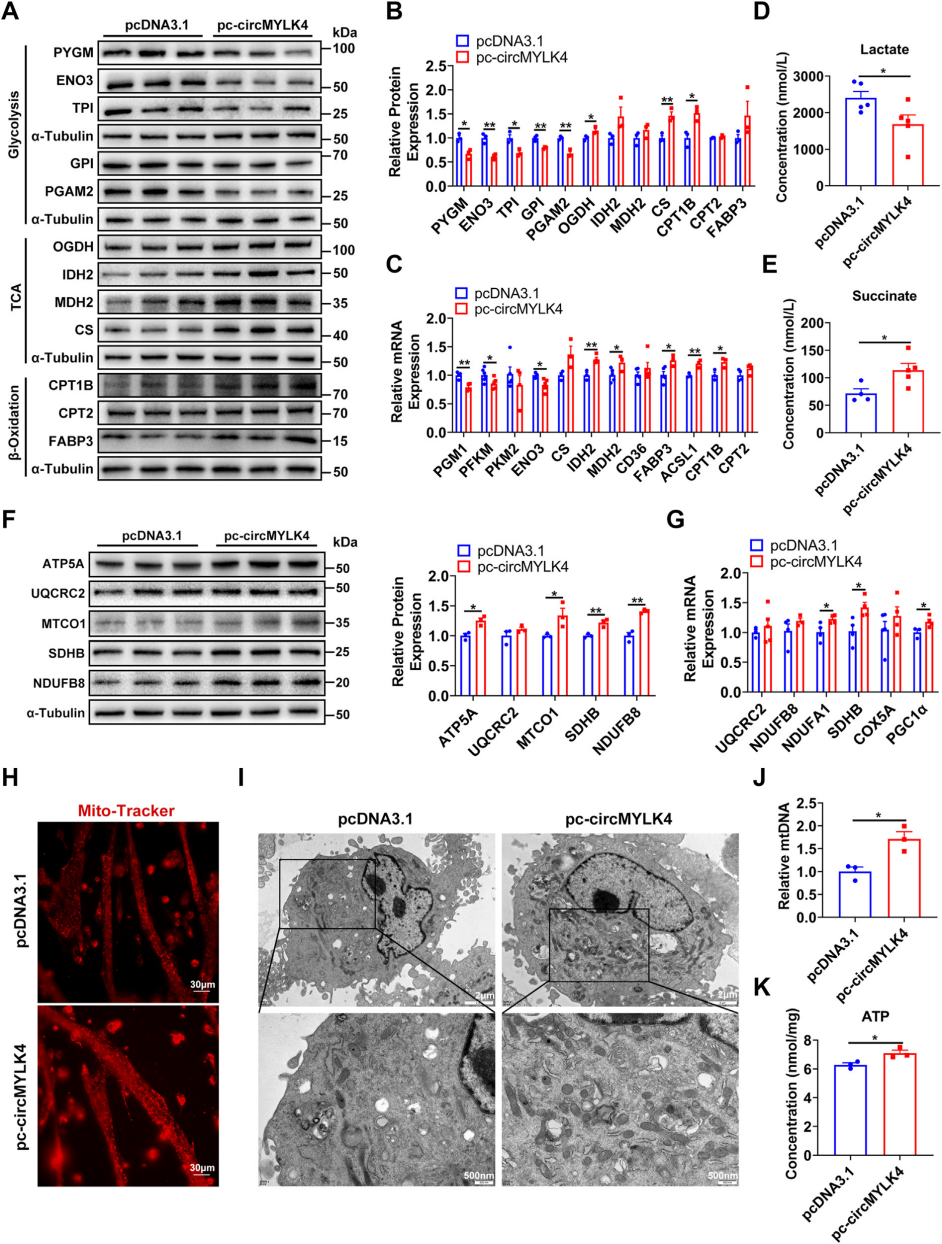
**Overexpression of circMYLK4 induces metabolic reprogramming in skeletal muscle satellite cells.***A*, Western blot analysis of protein markers for glycolysis, TCA cycle, and fatty acid β-oxidation in skeletal muscle satellite cells transfected with circMYLK4 overexpression plasmid. *B*, quantification of relative protein levels of glycolysis, TCA cycle, and fatty acid β-oxidation protein markers in skeletal muscle satellite cells transfected with circMYLK4 overexpression plasmid. *C*, mRNA expression levels of key genes involved in glycolysis, TCA cycle, and fatty acid β-oxidation in skeletal muscle satellite cells transfected with circMYLK4 overexpression plasmid. *D*, changes in lactate levels in skeletal muscle satellite cells overexpressing circMYLK4. *E*, changes in succinate levels in skeletal muscle satellite cells overexpressing circMYLK4. *F*, Western blot analysis and quantification of relative protein levels of electron transport chain (ETC) complex protein markers in skeletal muscle satellite cells transfected with circMYLK4 overexpression plasmid. *G*, mRNA expression levels of key genes involved in mitochondrial function in skeletal muscle satellite cells transfected with circMYLK4 overexpression plasmid. *H*, MitoTracker staining of skeletal muscle satellite cells overexpressing circMYLK4. *I*, electron microscopy observation of mitochondria in skeletal muscle satellite cells overexpressing circMYLK4. *J*, quantitative measurement of mitochondrial DNA content in skeletal muscle satellite cells overexpressing circMYLK4. *K*, ATP content in skeletal muscle satellite cells overexpressing circMYLK4. Data are mean ± SEM of three independent experiments (∗*p* < 0.05, ∗∗*p* < 0.01).

To further investigate the effect of circMYLK4 on skeletal muscle metabolism, we overexpressed circMYLK4 in porcine skeletal muscle satellite cells. As expected, the glycogen content of the circMYLK4 overexpression group was relatively higher. (Fig. S6*B*). We found that overexpression of circMYLK4 significantly decreased the protein levels of glycolytic genes PYGM, ENO3, TPI, GPI, and PGAM2, and significantly increased the protein levels of OGDH, CS in the TCA cycle, and CPT1B in fatty acid β-oxidation (Fig. 5, *A* and *B*). Similarly, circMYLK4 significantly suppressed the mRNA levels of glycolytic genes *PGM1*, *PFKM*, and *ENO3*, and significantly increased the mRNA levels of *IDH2*, *MDH2* in the TCA cycle, and *FABP3*, *ACSL1*, and *CPT1B* in fatty acid β-oxidation (Fig. 5*C*). In addition, we measured the concentrations of lactate and succinate in cells and found that circMYLK4 significantly decreased the concentration of lactate and increased the concentration of succinate (Fig. 5, *D* and *E*). It shows that the glycolytic ability of the cells is weakened and the oxidation ability is enhanced after overexpression of circMYLK4. Next, we detected the expression of mitochondria-related genes and found that circMYLK4 significantly increased the protein levels of mitochondrial complexes ATP5A, MTCO1, SDHB, and NDUFB8, and the mRNA levels of mitochondria-related genes *NDUFA1*, *SDHB*, and *PGC1α* (Fig. 5, *F* and *G*). Mitochondria staining with MitoTracker red revealed a significant increase in mitochondria area in circMYLK4 myotubes as compared to control myotubes (Fig. 5*H*). As expected, transmission electron microscopy showed that circMYLK4 significantly increased the number of mitochondria (Fig. 5*I*). Similarly, circMYLK4 significantly increased the mitochondrial copy number (Fig. 5*J*). A change in the type of metabolism means a change in the synthesis of ATP in the cell. We found that circMYLK4 significantly increased ATP content in cells (Fig. 5*K*). The above results indicated that circMYLK4 inhibited the glycolysis process of muscle satellite cells and increased fatty acid oxidation and mitochondrial oxidative phosphorylation.

### CACNA2D2 was identified as a circMYLK4 binding protein

**Figure 6:**
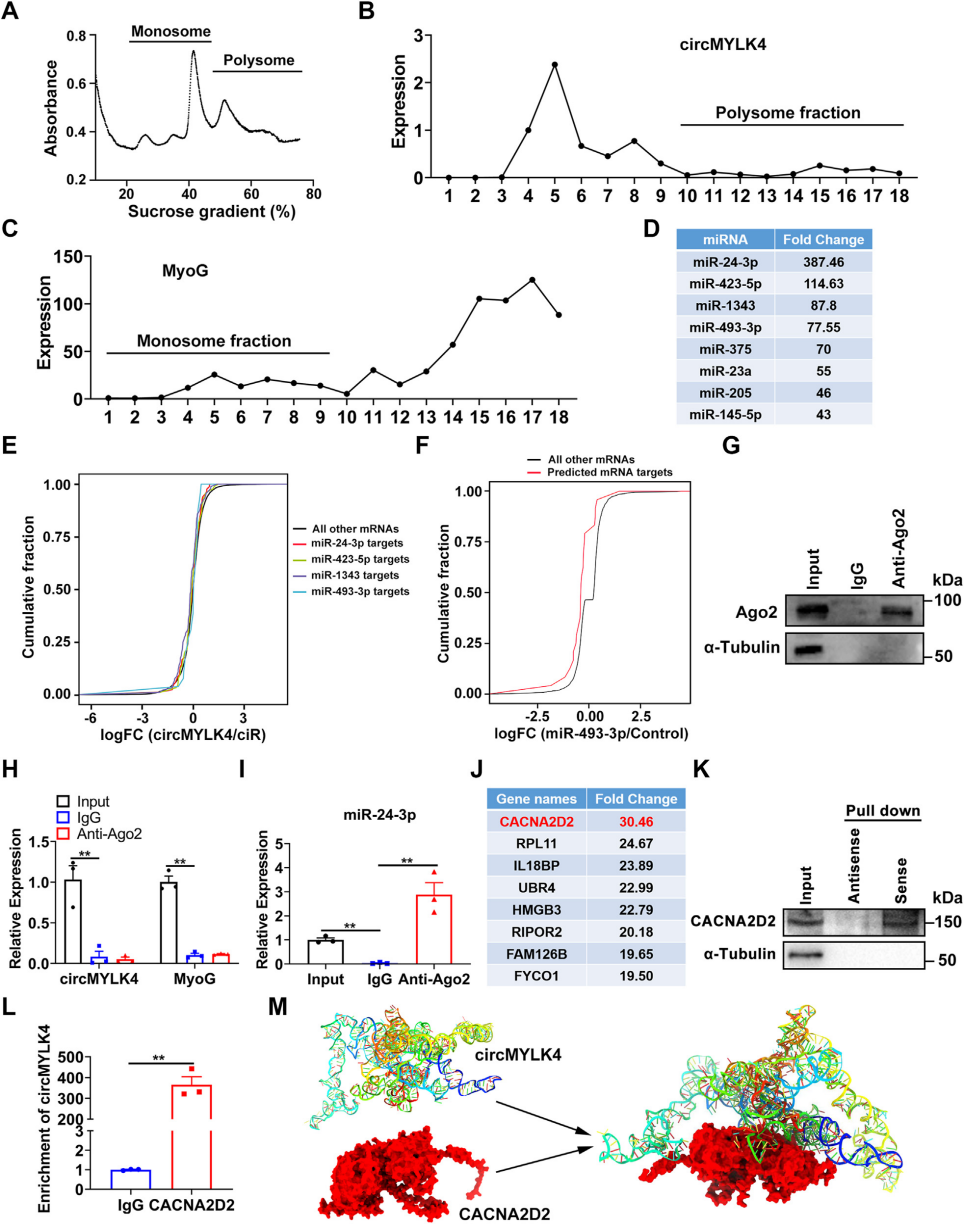
**circMYLK4 interacts with CACNA2D2 protein.***A*, a representative polysome profile of skeletal muscle satellite cells overexpressing circMYLK4. *B*, the transcript distribution of circMYLK4 across the different polysome fractions. *C*, the transcript distribution of MyoG across the different polysome fractions. *D*, identification of the top eight miRNAs binding to circMYLK4 through RNA pull-down assay. *E*, representative cumulative distribution curves of mRNA targets predicted to bind with four miRNAs (color) that interact with circMYLK4, compared to the reference curve (*black*). *F*, cumulative distribution curve of log2 RNA-seq fold changes in miR-493-3p mRNA targets (*red*), compared with reference curve of all other detected genes (*black*). *G*, AGO2 was precipitated from skeletal muscle satellite cells lysate by anti-AGO2 in RNA immunoprecipitation assay and detected by Western blot. *H*, expression of circMYLK4 and MyoG in samples immunoprecipitated with IgG and anti-AGO2. *I*, expression of miR-24-3p in samples immunoprecipitated with IgG and anti-AGO2. *J*, identification of the top eight proteins binding to circMYLK4 through RNA pull-down assay. *K*, representative western blots of CACNA2D2 pulled down by circMYLK4. *L*, expression of circMYLK4 in samples immunoprecipitated with IgG and anti-CACNA2D2. *M*, three-dimensional simulated structure of the binding between circMYLK4 and CACNA2D2. Data are mean ± SEM of three independent experiments (∗∗*p* < 0.01).

To identify the molecular mechanism that drives circMYLK4 to alter skeletal muscle metabolism. We first determined the localization of circMYLK4 and found that circMYLK4 was mainly located in the cytoplasm (Fig. S5*A*). It is implied that circMYLK4 may act at a post-transcriptional level. Previous studies have revealed that circRNA can encode peptides, act as molecular sponges for miRNAs, or bind proteins, thereby regulating the expression of target genes. We predict that circMYLK4 contains three putative open reading frames (ORF) that can be potentially translated at the same start codon as the canonical protein sequence (Fig. S5*B*). To test the protein-coding ability of circMYLK4, we performed polysome profiling in skeletal muscle satellite cells to examine whether circMYL4 is associated with polysomes (Fig. 6*A*). We found that circMYLK4 is exclusively present in the monosome fraction (Fig. 6*B*), while MyoG is present in the polysome fraction (Fig. 6*C*), indicating that circMYLK4 is not translated. In order to clarify the miRNAs adsorbed by circMYLK4, we performed miRNA sequencing analysis on the RNA pull-down experiment results and selected 217 differentially expressed miRNAs. Some of the miRNAs are shown in Figure 6*D*. We performed binding site prediction on the four miRNAs with the greatest differences and validated their binding to circMYLK4 through dual-luciferase assays (Fig. S5, *C*–*F*). The results indicated that miR-24-3p, miR-423-5p, miR-1343, and miR-493 were unable to bind to circMYLK4 (Fig. S5, *G*–*J*). To verify our results, we analyzed the target genes of miR-493-3p from previous studies, and observed the expected leftward shift in the cumulative distribution curve of its mRNA targets (Fig. 6*F*), demonstrating the repressive effect of miR-493-3p on these mRNAs. However, overexpression of circMYLK4 did not cause any significant shift in the distribution of mRNA targets for all putatively binding miRNAs (Fig. 6*E*). Furthermore, to demonstrate the function of circRNA as a miRNA sponge, it is necessary to provide evidence of the binding interaction between circRNA and the core protein Argonaute-2 (AGO2) of the RNA-induced silencing complex (RISC) (32). We conducted RNA immunoprecipitation (RIP) with AGO2 antibody (Fig. 6*G*), followed by RT-qPCR to examine whether AGO2 could pull down circMYLK4. Although AGO2 could retrieve several detected miR-24-3p as expected (Fig. 6*I*), it did not retrieve circMYLK4 and MyoG (Fig. 6*H*), suggesting no interaction between the two. Taken together, we conclude that circMYLK4 is unlikely to act as a regulator of skeletal muscle metabolism through sponge miRNAs.

Next, we performed mass spectrometry analysis on the RNA pull-down product to identify the protein binding to circMYLK4. We screened 50 differentially expressed proteins, and the top eight proteins with the most significant differences are shown in Figure 6*J*. Through Figure 4, *B* and *D*, we observed that after overexpression of circMYLK4, both upregulated and downregulated differentially expressed genes were enriched in the calcium signaling pathway. We focused our attention on CACNA2D2, which serves as an auxiliary subunit of dihydropyridine receptors and regulates intracellular calcium levels (33, 34). Then, we conducted the RNA pull-down assay and used the precipitated protein for Western blot analysis. Among them, we found that circMYLK4 could specifically bind to CACNA2D2 (Fig. 6*K*). Furthermore, we carried out RIP with a CACNA2D2 antibody and confirmed the interaction between CACNA2D2 and circMYLK4 in muscle satellite cells (Fig. 6*L*). Three-dimensional structures of RNAs are the basis of their biological functions (35). We predicted the tertiary structure of circMYLK4 using 3dRNA (http://biophy.hust.edu.cn/new/3dRNA). The tertiary structure of CACNA2D2 was then downloaded from the AlphaFold protein structure database (https://alphafold.ebi.ac.uk/entry/A0A286ZNE8). The binding of circMYLK4 to CACNA2D2 was simulated using HDOCK SERVER (http://hdock.phys.hust.edu.cn/) (Fig. 6*M*). In general, circMYLK4 exhibits a certain degree of specificity in its mechanism of action. It does not encode peptides or sequester miRNAs but rather exerts its function through binding to the protein CACNA2D2.

### circMYLK4 inhibits glycogen breakdown by binding to CACNA2D2

**Figure 7:**
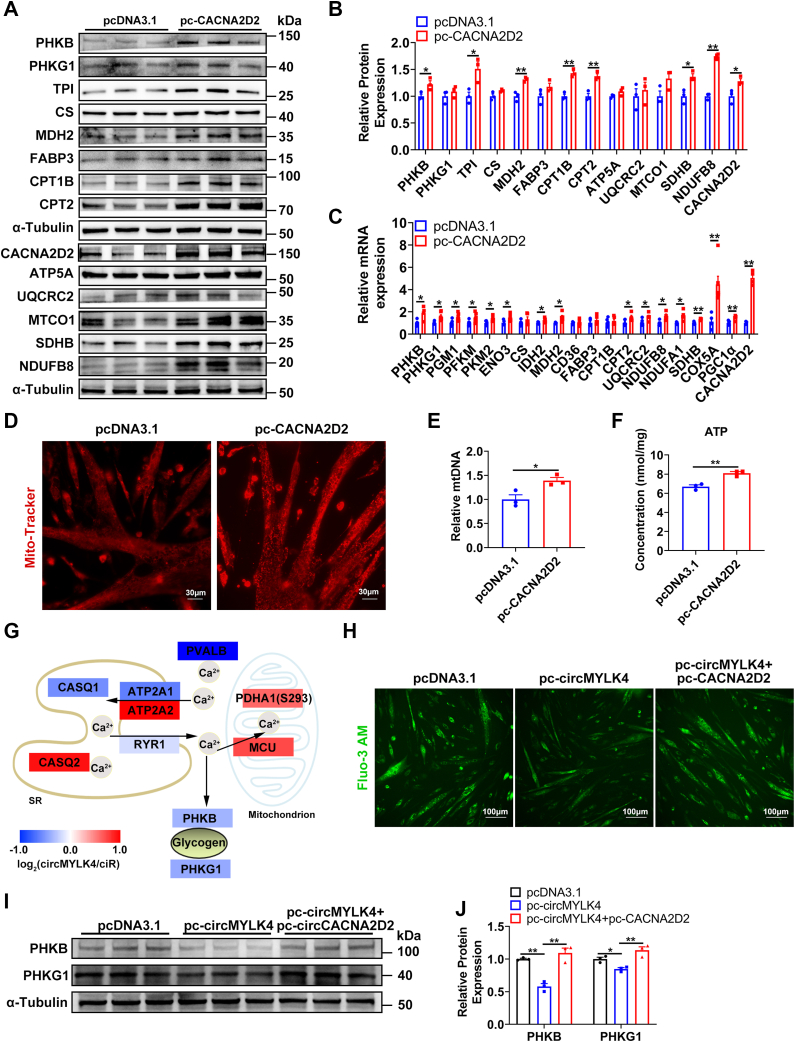
**circMYLK4 inhibits glycogen breakdown by binding to CACNA2D2.***A*, Western blot analysis of protein markers for glycolysis, TCA cycle, fatty acid β-oxidation, and ETC complex in skeletal muscle satellite cells transfected with CACNA2D2 overexpression plasmid. *B*, quantification of relative protein levels of glycolysis, TCA cycle, fatty acid β-oxidation, and ETC complex protein markers in skeletal muscle satellite cells transfected with CACNA2D2 overexpression plasmid. *C*, mRNA expression levels of key genes involved in glycolysis, TCA cycle, fatty acid β-oxidation, and OXPHOS in skeletal muscle satellite cells transfected with CACNA2D2 overexpression plasmid. *D*, MitoTracker staining of skeletal muscle satellite cells overexpressing CACNA2D2. *E*, quantitative measurement of mitochondrial DNA content in skeletal muscle satellite cells overexpressing CACNA2D2. *F*, ATP content in skeletal muscle satellite cells overexpressing CACNA2D2. *G*, mapping of protein abundance changes in proteins involved in intracellular calcium homeostasis between the ciR and circMYLK4 porcine skeletal muscle. *H*, assessment of intracellular Ca+ levels based on calcium ion fluorescent probes. *I*, protein expression levels of key glycogenolytic kinases after co-transfection of circMYLK4 and CACNA2D2. *J*, quantification of protein levels of key glycogenolytic kinases after co-transfection of circMYLK4 and CACNA2D2. Data are mean ± SEM of three independent experiments (∗*p* < 0.05, ∗∗*p* < 0.01).

To clarify the effect of CACNA2D2 on skeletal muscle metabolism, we overexpressed CACNA2D2 in porcine skeletal muscle satellite cells. The initial increase in glycogen breakdown is due to the posttranslational modification of glycogen phosphorylase (PYGM) from a less active *b* form to the constitutively active *a* form by phosphorylase kinase (PHK) (36). We found that overexpression of CACNA2D2 significantly promoted the protein expression of PHKB (Fig. 7, *A* and *B*), indicating that it promoted the breakdown of glycogen. Additionally, CACNA2D2 significantly promoted the protein expression of glycolysis gene TPI, TCA cycle gene MDH2, fatty acid oxidation genes CPT1B and CPT2, oxidative phosphorylation genes SDHB and NDUFB8 (Fig. 7, *A* and *B*). mRNA levels also indicate that CACNA2D2 promotes glycogen breakdown, glycolysis, fatty acid oxidation, TCA cycle, and oxidative phosphorylation (Fig. 7*C*). MitoTracker staining revealed that overexpression of CACNA2D2 significantly increased the mitochondrial area (Fig. 7*D*). Similarly, CACNA2D2 increased the mitochondrial copy number (Fig. 7*E*). The ATP levels in the cytoplasm also showed a significant increase (Fig. 7*F*).

CACNA2D2 primarily exerts its function by influencing the calcium ion levels in the cytoplasm (37). We analyzed the proteomic profiling of calcium ion pathway-related proteins after circMYLK4 overexpression. It was found that circMYLK4 significantly reduced the protein level of RYR1 and the calcium-binding protein PVALB (Fig. 7*G*). The decrease in cytoplasmic calcium ion levels after overexpression of circMYLK4 resulted in a significant reduction in the protein levels of the key enzymes PHKB and PHKG1 involved in glycogen breakdown (Fig. 7*G*). However, the protein levels of mitochondrial calcium ion channel protein MCU and the phosphorylation levels of the key enzyme PDHA1 involved in catalyzing pyruvate oxidation were significantly increased (Fig. 7*G*). Additionally, we utilized Fluo-3 AM to measure the cytoplasmic calcium ion concentration and found that circMYLK4 significantly reduced the levels of cytoplasmic calcium ions. This inhibitory effect was restored upon co-transfection with CACNA2D2 (Fig. 7*H*). Similarly, the inhibitory effect of circMYLK4 on glycogen breakdown was also restored upon co-transfection with CACNA2D2 (Fig. 7, *I* and *J*). In summary, circMYLK4, through its interaction with CACNA2D2, decreases the cytoplasmic calcium ion levels, resulting in the inhibition of glycogen breakdown and subsequently affecting the glycolysis process.

## Discussion

In our previous study, we identified a novel circRNA, circMYLK4, which exhibits high expression in slow-twitch skeletal muscle fibers. Here, we elucidated the role and mechanism of circMYLK4 in skeletal muscle metabolism and mitochondrial function. Specifically, we found that circMYLK4 inhibits glycolysis, promotes fatty acid β-oxidation and the TCA cycle, and enhances mitochondrial function. Mechanistically, circMYLK4 binds to the voltage-gated calcium channel subunit CACNA2D2, inhibiting the release of calcium ions from the sarcoplasmic reticulum, leading to a decrease in cytosolic calcium concentration and subsequent inhibition of glycogen breakdown and glycolysis. The increased fatty acid β-oxidation enhances mitochondrial function. These findings highlight the indispensable role of circMYLK4, which affects the balance between glycolysis and OXPHOS, in skeletal muscle energy metabolism.

The majority of cells utilize two main pathways to generate ATP: glycolysis and OXPHOS. Studies have shown that circRNA can affect the balance of cellular energy metabolism. For instance, circACC1 stabilizes and enhances the enzymatic activity of the AMPK holoenzyme by forming a ternary complex with the β and γ subunits of AMPK, thereby accelerating fatty acid β-oxidation and glycolysis (38). circPUM1 derived from the mitochondrial nuclear genome can interact with UQCRC2, the core protein of mitochondrial complex Ⅲ, to regulate mitochondrial oxidative phosphorylation (39). PKM2 promotes lactate production in cells by inducing glycolysis. circSRRM4 combines with and inhibits serine and arginine-rich splicing factor 3 (SRSF3) from joining the ubiquitin-proteasome pathway, improving the SRSF3-regulated alternative splicing of PKM, and consequently stimulating glycolysis in cells (40). We have discovered that circMYLK4 inhibits the glycolytic process and promotes fatty acid oxidation and OXPHOS in skeletal muscle. Despite being different cell types, they all utilize a balance between glycolysis and oxidative phosphorylation (OXPHOS) to meet their individual energy demands.

In this experiment, we employed a comprehensive analysis combining transcriptomics, proteomics, and phosphoproteomics to elucidate the function of circMYLK4. We identified not only muscle fiber type and energy metabolism marker genes but also several genes associated with skeletal muscle function. For example, the deletion of C18ORF25 leads to reduced muscle fiber size, contractile force, and exercise capacity, which may be attributed to impaired SR Ca^2+^ pump function. Conversely, phosphorylation of S67 on C18ORF25 enhances the generation of contractile force (41). In this study, overexpression of circMYLK4 increased the phosphorylation level of C18ORF25 S67. The effect of circMYLK4 on muscle contraction was consistent with the phenotype of C18ORF25, and both circMYLK4 and C18ORF25 affected the intracellular Ca^2+^ concentration. The absence of ANKRD2 in slow-twitch muscle fibers results in adverse effects on the expression of myogenic program-associated cytokines in slow-twitch muscle fibers (42), suggesting that ANKRD2 is essential for normal slow-twitch contraction. The overexpression of circMYLK4 significantly enhanced the protein expression of ANKRD2 and upregulated the expression of slow muscle-related genes, which is consistent with the findings reported above. Do those differentially expressed but uncharacterized proteins or phosphorylation sites also play important roles in skeletal muscle development and energy metabolism, warranting further screening and investigation.

The primary function of cytoplasmic localized circRNAs is to serve as miRNA sponges, interacting with proteins or acting as templates for protein translation (43). circUBE3A regulates myoblast proliferation and differentiation by targeting miR-28-5p (44). On the other hand, circTmeff1 promotes muscle atrophy by interacting with TDP-43 and encoding the TMEFF1-339aa protein (45). In this experiment, we proved that circMYLK4 cannot act as a miRNA sponge through RNA pull-down assays, dual-luciferase assays combined with cumulative distribution curves, and AGO2-RIP assays. In addition, although circMYLK4 has three potential open reading frames, the polysome profiling assay showed that circMYLK4 has no coding ability. However, RNA pull-down assay combined with mass spectrometry and RIP assay proved that circMYLK4 could bind to the protein CACNA2D2. This indicates that the function and molecular mechanism of circMYLK4 are highly specific. In fact, it is not uncommon to find that circRNA functions by binding to proteins rather than by adsorbing miRNA and coding proteins (46, 47). circArhgap5-2 neither sponges miRNAs nor encodes novel peptides, suggesting that it likely functions by binding proteins to regulate adipogenesis (46). circTFDP2 lacks IRES or ORF and does not interact with the AGO2 protein, suggesting its low protein-coding potential and inability to function as a miRNA sponge (47).

Skeletal muscle cells undergo membrane potential changes in response to excitatory signals. Voltage-gated calcium channels interact with the RyR1 protein on the sarcoplasmic reticulum membrane upon sensing the membrane potential changes. This interaction leads to the rapid and extensive release of Ca^2+^ from the sarcoplasmic reticulum (SR) into the cytoplasm by RyR1, thereby triggering skeletal muscle contraction (48). CACNA2D2 serves as an auxiliary subunit of voltage-gated calcium channels, participating in the regulation of intracellular calcium ion concentration (33, 34). We found that circMYLK4 can interact with CACNA2D2 and influence its function, leading to a decrease in intracellular calcium ion concentration. However, it remains unclear how the binding of circMYLK4 to CACNA2D2 affects the interaction between calcium channels and RyR1. Oxidation, phosphorylation, and nitrosylation of RyR can result in the leakage of calcium ions from the sarcoplasmic reticulum (49). Prolonged leakage leads to excessive elevation of cytosolic and mitochondrial calcium ions, resulting in increased ROS production, mitochondrial dysfunction, and cellular damage (50). However, acute RyR1 calcium ion leakage in muscle is believed to be beneficial, as it increases mitochondrial calcium uptake and enhances mitochondrial oxidative phosphorylation and NADH-related mitochondrial respiratory capacity (51). In our findings, while the binding of circMYLK4 to CACNA2D2 inhibited the release of calcium ions from SR, mitochondrial function was actually enhanced. This leads us to speculate whether sustained inhibition of calcium release from SR would result in impaired mitochondrial function, which is worth further consideration.

**Figure 8:**
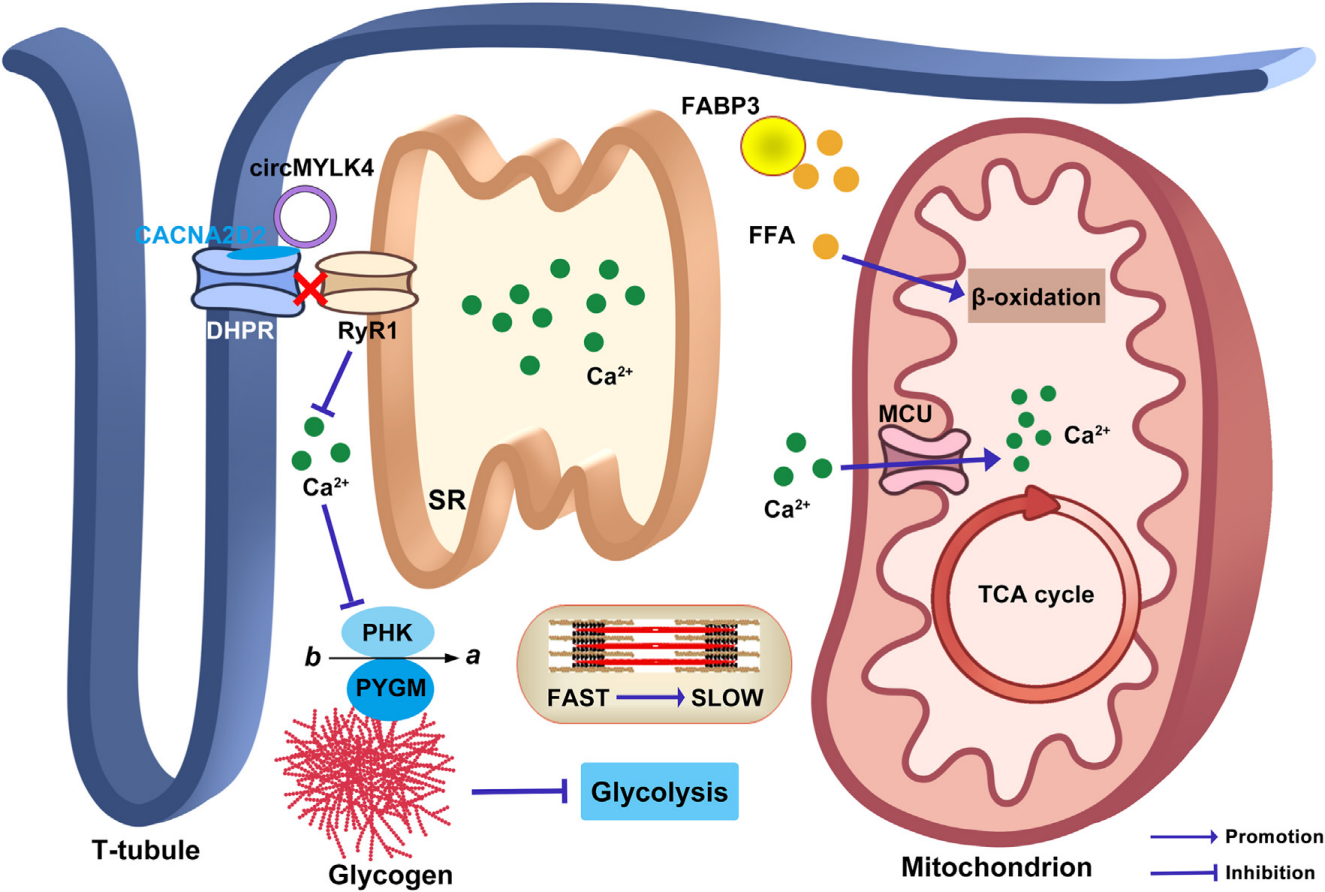
**Schematic model of the mechanism of action of circMYLK4.** circMYLK4 binding to the auxiliary subunit of DHPR affects the interaction between DHPR and RyR1, resulting in reduced Ca^2+^ release from the SR. The decrease in cytoplasmic Ca^2+^ concentration inhibits glycogen breakdown, thereby suppressing glycolysis. However, the increased fatty acid β-oxidation enhances mitochondrial OXPHOS. These processes are accompanied by the transition of glycolytic fibers to oxidative fibers.

In conclusion, our results indicate that circMYLK4 can induce a metabolic shift in skeletal muscle from glycolysis to oxidative phosphorylation. Mechanistically, the binding of circMYLK4 to CACNA2D2 inhibits the release of Ca^2+^ from the sarcoplasmic reticulum, leading to suppressed glycogen breakdown and subsequently affecting glycolytic processes. On the other hand, the increased fatty acid β-oxidation enhances mitochondrial function. These processes are accompanied by the transition of glycolytic fibers to oxidative fibers (Fig. 8). In general, these findings suggest that circMYLK4 plays a significant role in maintaining the metabolic homeostasis of skeletal muscle.

## Experimental procedures

### Animals and animal care

C57BL/6 male mice were obtained from the Animal Center of the Fourth Military Medical University. The 3-day-old piglets were obtained from the Animal Experimental Station of Northwest A&F University. All animal surgeries were performed in compliance with the ARRIVE guidelines. All animal care and sample collection procedures were strictly performed in accordance with the approved protocols of the Experimental Animal Management and Ethical Review Committee of Northwest A&F University (NWAFU-314020038).

### *In vivo* injection of circMYLK4-AAV

The overexpression vector of circMYLK4 and viral packaging were completed by Guangzhou geneseed. The packaged circMYLK4-AAV virus had a titer of 10^∧^13 GC/ml. Twenty 8-week-old mice were selected for gastrocnemius injection of circMYLK4-AAV and control virus, respectively (29). Sample collection was carried out 4 weeks after the injections.

### H&E and immunofluorescence staining

The gastrocnemius muscle was fixed with 4% paraformaldehyde (PFA) and embedded in paraffin. Tissue samples were sectioned at a thickness of 6 μm and stained with hematoxylin-Eosin (HE). For immunofluorescence staining, the entire muscle tissue was immediately frozen in OCT and then sectioned into 10 μm thick cross-sections using a cryostat. The cross-sections were fixed in 4% PFA for 15 min and quenched with 100 mM glycine for 10 min. The fixed tissue was incubated with a blocking buffer for 2 h. Subsequently, the tissue sections were incubated overnight at 4 °C with the primary antibody diluted in blocking solution, followed by incubation with the secondary antibody at room temperature for 1 h.

### Proteomics, phosphoproteomics, and PRM-targeted quantification

After thoroughly grinding the samples to a powder with liquid nitrogen, they were added to four times the volume of lysis buffer and sonicated for cell lysis. The supernatant was collected after centrifugation at 4 °C and 12,000*g* for 10 min for protein concentration determination. An equal amount of protein was slowly added to 20% TCA, vortexed, and then precipitated at 4 °C for 2 h. The supernatant was discarded after centrifugation at 4500*g* for 5 min, and the pellet was washed with pre-chilled acetone, air-dried, and then resuspended in 200 mM TEAB. The resuspended pellet was sonicated and digested overnight with trypsin. The protein solution was reduced with 5 mM DTT at 56 °C for 30 min, followed by alkylation with IAA at room temperature in the dark for 15 min. The peptide mixture was dissolved in 0.1% formic acid and separated using an EASY-nLC 1200 ultra-high-performance liquid chromatography system. After peptide separation, they were ionized in the NSI ion source and analyzed using a Q Exactive HF-X mass spectrometer. For phosphorylation analysis, prior to peptide separation, the peptides were dissolved in an enrichment buffer solution (50% acetonitrile/0.5% acetic acid) and incubated with IMAC material. Subsequently, the material was washed three times with buffer solutions containing 50% acetonitrile/0.5% acetic acid and 30% acetonitrile/0.1% trifluoroacetic acid, and phosphopeptides were eluted with 10% ammonia solution. The eluate was vacuum-dried and desalted for liquid chromatography-mass spectrometry analysis.

### RNA sequencing

After total RNA extraction using the Trizol method, the integrity of the RNA samples and the presence of DNA contamination were analyzed using agarose gel electrophoresis. The purity of the RNA was assessed using a NanoPhotometer spectrophotometer, and the RNA concentration was quantified using the Qubit 2.0 Fluorometer. The RNA integrity was evaluated using the Agilent 2100 Bioanalyzer. The NEBNext UltraTM RNA Library Prep Kit was used for library construction. mRNA with poly(A) tails was enriched using oligo(dT) magnetic beads, followed by random fragmentation of the obtained mRNA using divalent cations in NEB Fragmentation Buffer. Using the fragmented mRNA as a template, the first strand of cDNA was synthesized using random oligonucleotides as primers in the presence of M-MuLV reverse transcriptase. The RNA chain was then degraded using RNaseH, and the second strand of cDNA was synthesized using DNA polymerase I and dNTPs. The purified double-stranded cDNA was subjected to end repair, A-tailing, and ligation of sequencing adapters. The cDNA fragments of approximately 200 bp were selected using AMPure XP beads, followed by PCR amplification and purification of the PCR products using AMPure XP beads to obtain the final library. After library construction, the library was initially quantified using the Qubit 2.0 Fluorometer, followed by the assessment of the insert size of the library using the Agilent 2100 Bioanalyzer. Once the library passed quality control, it was subjected to sequencing on the appropriate platform. The raw data obtained from sequencing were filtered using fastp v0.19.3 to remove any artifacts or low-quality reads.

### Bioinformatics analysis

The transcriptome data underwent quality control using fastp with the parameters --n_base_limit set to 15 and --Qualified_Quality_phred set to 20. Aligned genomes were obtained using hisat2 with default parameters. Differential expression analysis was performed using DESeq2 and edgeR. MaxQuant was utilized for analyzing the mass spectrometry data of proteome and phosphoproteome. Principal component analysis was conducted on all identified features to explore the primary source of variation within each omics dataset. Volcano maps and heat maps were employed for visualizing the differential multi-omics data. The volcano map, drawn by PTM cloud platform, had a fold change threshold of 1.2, while the heat map was generated using TBtools software. Metascape (v3.5) facilitated comprehensive analysis of multi-omics datasets, while Circos visualized correlations between features from different clusters or their annotations. Enrichment clustering based on integrated GO and KEGG sources was applied in metascape, followed by visualization of relevant data networks using Cytoscape.

### Metabolomics

Approximately 0.05 g of the sample was mixed with 500 μl of 70% methanol and vortexed at 2500 rpm for 3 min. The mixture was then centrifuged at 4 °C and 12,000 rpm for 10 min. Next, 300 μl of the supernatant was transferred to a −20 °C freezer and left for 30 min. Afterward, it was centrifuged at 4 °C and 12,000 rpm for 10 min. Following centrifugation, 200 μl of the supernatant was transferred to a protein precipitation plate for LC-MS analysis.

### Isolation, culture, and transfection of skeletal muscle satellite cells

Muscle satellite cells were isolated from the hind limb skeletal muscles of 3-day-old pigs. The muscle tissue was minced and digested separately with type Ⅱ collagenase (17101015, Gibco) and trypsin (25200072, Gibco). The digestion was neutralized in RPMI 1640 medium (11875093, Gibco) containing 20% fetal bovine serum (SH30084.03HI, HyClone), 1% penicillin-streptomycin (P1400, Solarbio), 1% chicken embryo extract (abs80002, Absin), and 1% FGFb (GMP-C046, Novoprotein). The primary cells were purified using the differential adhesion method. For differentiation, myoblast cells were seeded on culture plates coated with matrix gel and differentiated in DMEM (G4510, Servicebio) containing 2% horse serum (S9050, Solarbio) and 1% penicillin-streptomycin. For transfection, pcDNA3.1-circMYLK4 plasmid and pcDNA3.1-CACNA2D2 plasmid were mixed with lipofectamine 2000 Reagent (11668019, Invitrogen) in Opti-MEM (31985070, Gibco) media and transfected into cells following the protocol from manufacture.

### RNA extraction and real-time qPCR

Total RNA was extracted from cells and tissues using the AG RNAex Pro reagent (AG21102, Accurate Biology) following the manufacturer's instructions. cDNA synthesis was performed using a reverse transcription kit (R323-01 and R312-02, Vazyme) according to the standard protocol. Quantitative experiments were conducted using the ChamQ SYBR qPCR Master Mix (Q311-02, Vazyme), with 18s rRNA selected as the reference gene. The relative RNA levels were determined using the 2^−ΔΔCt^ method. miRNA primers were purchased from Ribobio (Guangzhou, China). The primer sequences of genes detected in this study are listed in Table 1.

**Table 1:** The primer sequences for real-time qPCR

Genes	Forward	Reverse
*PGM1*	GATCCTGTGGACGGAAGCAT	AGGTGATAACAGTGGGTGCG
*PFKM*	GGAGAGCTGAGACTATAAGAGTGG	CCAGAGGTTAACACGGCGAT
*PKM2*	CCACCGCAAGCTGTTTGAAG	TAGCCACCTGATGTGCAGAC
*ENO3*	GCCACCAATGTGGGAGATGA	CTTCCCGTTGCGGTAGAACT
*CS*	CTCTTCGGAGCCAAGAATGC	TGCCTCTCATGCCACCATAC
*IDH2*	CTTGGCCTGATGACGTCTGT	GTCATGGCTCCGCTCTCTAC
*MDH2*	AATGCCAAGGTAGCTGTGCT	CCTTTCACAGTCGCTCTGGT
*CD36*	TAGGAATCCCACTGCCTCAC	GCTTCAAGTGCTGGGTCAAA
*FABP3*	TTGTGACACTGGATGGAGGC	TAAGTGCGAGTGCAAACTGC
*ACSL1*	AGTCCTTCCTCCGATGATACTCT	GGACCACAGGGAAGATGGTG
*CPT1B*	TGGGGCTGGTCAATCACATC	GCCGTGCATCTCAAACATCC
*CPT2*	CTGACTGCTCGAAACCCCAT	GAACACTTCCGGCTCCAAGA
*UQCRC2*	CAACCCTGAGCAGCAAATACG	GTAACCAGAGCCACACCAGT
*NDUFB8*	GTGTAAGCACCTCTCCGGTT	TTCTTTGGTAGGATCGCCGC
*NDUFA1*	CGCGAGTCGGGACAAAAATC	CCTCAAAGGTGACCCGACTT
*SDHB*	ACAGCTCCCCGAATCAAGAA	CTCCGTTGATGTTCATGGCG
*COX5A*	CACCCGGCTTTGCCTTAGC	GCGAATTGACTGGATAGCGG
*PGC1α*	GTACCTCCGCCATCGAAGAA	CGAGCGCGGACGTCTTG
*circMYLK4*	GCGAGTAAAGCCCTGAAGCA	TCCTCTTGTCTTCGGCAGGT
*CACNA2D2*	GATGCCCTGTTAAGGCCACT	CACTGCTTCTGAGGCTGGTC
*MyoG*	ATGAGACATCCCCCTACTTCTACCA	GTCCCCAGCCCCTTATCTTCC
*PHKB*	TCAAACGACAAAGCAGCACC	TAAATGCAGCCCCGTAGCG
*PHKG1*	GACATAAGCGAGCCTTTCGG	ATGATCTTCACCGCGTACTCC
*MYH7*	AAGGGCTTGAACGAGGAGTAGA	TTATTCTGCTTCCTCCAAAGGG
*MYH2*	GCTGAGCGAGCTGAAATCC	ACTGAGACACCAGAGCTTCT
*MYH1*	AGAAGATCAACTGAGTGAACT	AGAGCTGAGAAACTAACGTG
*MYH4*	ATGAAGAGGAACCACATTA	TTATTGCCTCAGTAGCTTG
*18S*	CCCACGGAATCGAGAAAGAG	TTGACGGAAGGGCACCA

### Western blotting

Cellular total protein extraction was performed using radio-immunoprecipitation assay (RIPA) buffer (P0013B, Beyotime) supplemented with protease inhibitor (M7528, AbMole BioScience). Equal amounts of protein were separated by electrophoresis on 4 to 20% polyacrylamide gels (ET12420Gel, ACE Biotechnology), transblotted onto polyvinylidene fluoride (PVDF) membranes (IPVH00010, Millipore) and incubated with antibodies against MYH1 (1:50,000, 67299-1-lg, Proteintech), MYH2 (1:1000, SC-71, DSHB), MYH4 (1:1000, BF-F3, DSHB), MYH7 (1:1000, BA-D5, DSHB), PYGM (1:2000, 19716-1-AP, Proteintech), ENO3 (1:1000, PTM-6021, PTM BIO), TPI (1:1000, PTM-5901, PTM BIO), GPI (1:2000, PTM-5916, PTM BIO), PGAM2 (1:2000, PTM-6768, PTM BIO), OGDH (1:1000, PTM-5974, PTM BIO), IDH2 (1:1000,PTM-6878, PTM BIO), MDH2 (1:5000, PTM-7133, PTM BIO), CS (1:1000, CY6930, Abways), CPT1B (1:2000, 22170-1-AP, Proteintech), CPT2 (1:1000, 26555-1-AP, Proteintech), FABP3 (1:1000, PTM-6313, PTM BIO), ATP5A (1:1000, PTM-5163, PTM BIO), UQCRC2 (1:1000, PTM-5008, PTM BIO), MTCO1 (1:1000, PTM-5109, PTM BIO), SDHB (1:1000, PTM-5164, PTM BIO), NDUFB8 (1:1000, PTM-5899, PTM BIO), AGO2 (1:2000, 67934-1-lg, Proteintech), CACNA2D2 (1:1000, ab173293, abcam), PHKB (1:1000, 13400-1-AP, Proteintech), PHKG1 (1:2000, 16743-1-AP, Proteintech) or α-Tubulin (1:5000, PTM-5001, PTM BIO) at 4 °C overnight. Afterward, the membranes were incubated with HRP Conjugated AffiniPure Goat anti-mouse IgG (1:5000, BA1050, BOSTER) or anti-rabbit IgG (1:5000, BA1054, BOSTER) secondary antibodies for 1 h at room temperature. In certain experiments, a stripping buffer (CW0056, CWBIO) was used to remove the primary and secondary antibodies from the PVDF membrane for 15 min. After re-blocking the membrane, it was incubated overnight at 4 °C with another primary antibody. Subsequent steps were performed following the same procedure as mentioned above.

### Polysome profiling

Approximately 10^7^ cells were lysed using polysome profiling cell lysis buffer (Tris-HCl 25 mM pH 7.4, NaCl 150 mM, MgCl_2_ 5 mM, DTT 1 mM, PMSF 1 mM, RNase inhibitor 40 μ/ml, sodium deoxycholate 1%, NP40 0.5%). The cell lysate was incubated on ice for 30 min and then centrifuged at 4 °C, 13,000*g* for 10 min. The supernatant was collected and added to different density gradients of sucrose solution (10–45%) (Tris-HCl 25 mM pH 7.4, NaCl 150 mM, MgCl_2_ 5 mM, DTT 1 mM, PMSF 1 mM, RNase inhibitor 40 μ/ml, Cycloheximide 100 μg/ml). The mixture was subjected to ultracentrifugation at 36,000 rpm for 3 h, followed by fractionation and collection of each component. RNA from each fraction was isolated using Trizol.

### RNA pull-down assay

RNA pull-down experiments were performed using the Magnetic RNA-Protein Pull-Down Assay Kit (20164, Thermo Fisher Scientific). 10^7^ myoblast cells were fixed with 1% formaldehyde at room temperature for 30 min, followed by cell lysis using IP lysis buffer (87787, Thermo Fisher Scientific) at 4 °C for 2 h. The cell lysate was then hybridized overnight at 37 °C with a biotin-labeled oligo probe. Streptavidin magnetic beads were added to each binding reaction after washing the beads with IP lysis buffer five times. The products were detected using Western blotting or MS.

### RNA immunoprecipitation (RIP) assay

After washing with pre-chilled PBS, 10^7^ myoblast cells were lysed using IP lysis buffer (87787, Thermo Fisher Scientific) containing protease inhibitor (M7528, AbMole BioScience) and RNase inhibitor (N2511, Promega). The lysate was then incubated overnight at 4 °C with antibodies against AGO2 (1:100, 67934-1-lg, Proteintech), CACNA2D2 (1:100, ab173293, Abcam), control mouse IgG (1:100, 12-71, Millipore), or control rabbit IgG (1:100, 12-370, Millipore). Protein A/G magnetic beads (88802, Thermo Fisher Scientific) were added to each sample, and the mixture was incubated at 4 °C for 4 h. Following immunoprecipitation, RNA was extracted using Trizol and subjected to qRT-PCR experiments.

### Luciferase reporter assay

The binding sites of circMYLK4 with various miRNAs were synthesized by General Biology Systems Ltd. The 293T cell line (Stem Cell Bank, Chinese Academy of Science) was seeded in 48-well culture plates at a density of 8000 cells per well. When the cell density reached 70%, 250 ng of the binding sites were co-transfected with miRNA mimics or negative control (RiboBio) into the cells. After 24 h of transfection, the relative luciferase activities of Renilla and Firefly were measured using the dual-luciferase reporter system (F6075M, UElandy) according to the manufacturer’s protocol (US EVERBRIGHT).

### Glycogen assay

Glycogen content in cells and tissues was measured using the glycogen content kit (E−455-SH, Shanghai Hengyuan Biotechnology Co, LTD) according to the manufacturer's instructions. In brief, 0.1 to 0.2 g of tissue or 5 to 10 million cells were fully homogenized by adding 0.75 ml of extract. The mixture was thoroughly mixed in 95 °C water for 20 min. After cooling, the mixture was fixed to 5 ml with distilled water, mixed, centrifuged at 8000*g* for 10 min at 25 °C, and the supernatant was taken for measurement.

### Cytosolic Ca^2+^ imaging using Fluo-3 AM

Cellular calcium ion concentration was measured using the Fluo-3 AM calcium ion fluorescent probe (S1056, Beyotime). Cells were incubated at 37 °C in serum-free medium containing 5 μM Fluo-3 AM for 45 min. After washing, cells were further incubated in a normal culture medium for 20 min prior to microscopic observation.

### MitoTracker staining

Mitochondria were stained using MitoTracker Red CMXRos (40741ES50, YEASEN), a mitochondrial-specific red fluorescent probe. When the cells reached the desired density, the culture medium was aspirated, and pre-warmed MitoTracker Red CMXRos staining working solution at 37 °C was added. The cells were incubated under normal culture conditions for 30 min. The staining solution was then replaced with fresh culture medium, and the cells were observed under a fluorescence microscope.

### Mitochondrial morphological analysis

The cells were digested with trypsin at 1000 rpm for 5 min, and the supernatant was discarded after centrifugation. The cells were resuspended in 0.5% glutaraldehyde and incubated at 4 °C for 5 min. After centrifugation at 12,000 rpm for 10 min, the supernatant was discarded, and the cells were fixed with 3% glutaraldehyde followed by 1% osmium tetroxide fixation. Gradual dehydration was performed using acetone, and the samples were embedded in Ep812 resin. After sectioning, the slices were stained with uranyl acetate for 10 to 15 min, followed by lead citrate staining for 1 to 2 min. Finally, observation was conducted using a transmission electron microscope (JEM-1400FLASH, JEOL).

### Detection of intracellular ATP levels

The intracellular ATP levels were measured using an enhanced ATP assay kit (S0027, Beyotime). The lysed cells were centrifuged at 4 °C, 12,000*g* for 5 min, and the supernatant was collected. Prior to ATP detection, the detection solution was added to a 96-well plate and incubated at room temperature for 5 min. Then, the collected supernatant was added to the plate and mixed rapidly, and the relative luminescence units (RLU) were measured using a chemiluminescence analyzer.

### Statistical analysis

All data are presented as mean ± SEM. All quantitative analyses were performed using the Student’s *t* test and two-tailed distribution calculated with GraphPad Prism 8.0 (∗*p* < 0.05; ∗∗*p* < 0.01).

## Data availability

The raw data that supports the findings of this study are available from the corresponding author upon reasonable request.

## Supporting information

This article contains supporting information.

## Conflict of interest

The authors declare that they have no known competing financial interests or personal relationships that could have appeared to influence the work reported in this paper.
