# Supplementary material for: Circular RNA circMYLK4 shifts energy metabolism from glycolysis to OXPHOS by binding to the calcium channel auxiliary subunit CACNA2D2
Source: J Biol Chem. 2024 May 30;300(7):107426. doi: 10.1016/j.jbc.2024.107426 (PMC11245919; doi:10.1016/j.jbc.2024.107426)
Supplement: Supporting Figures [file mmc1.docx]

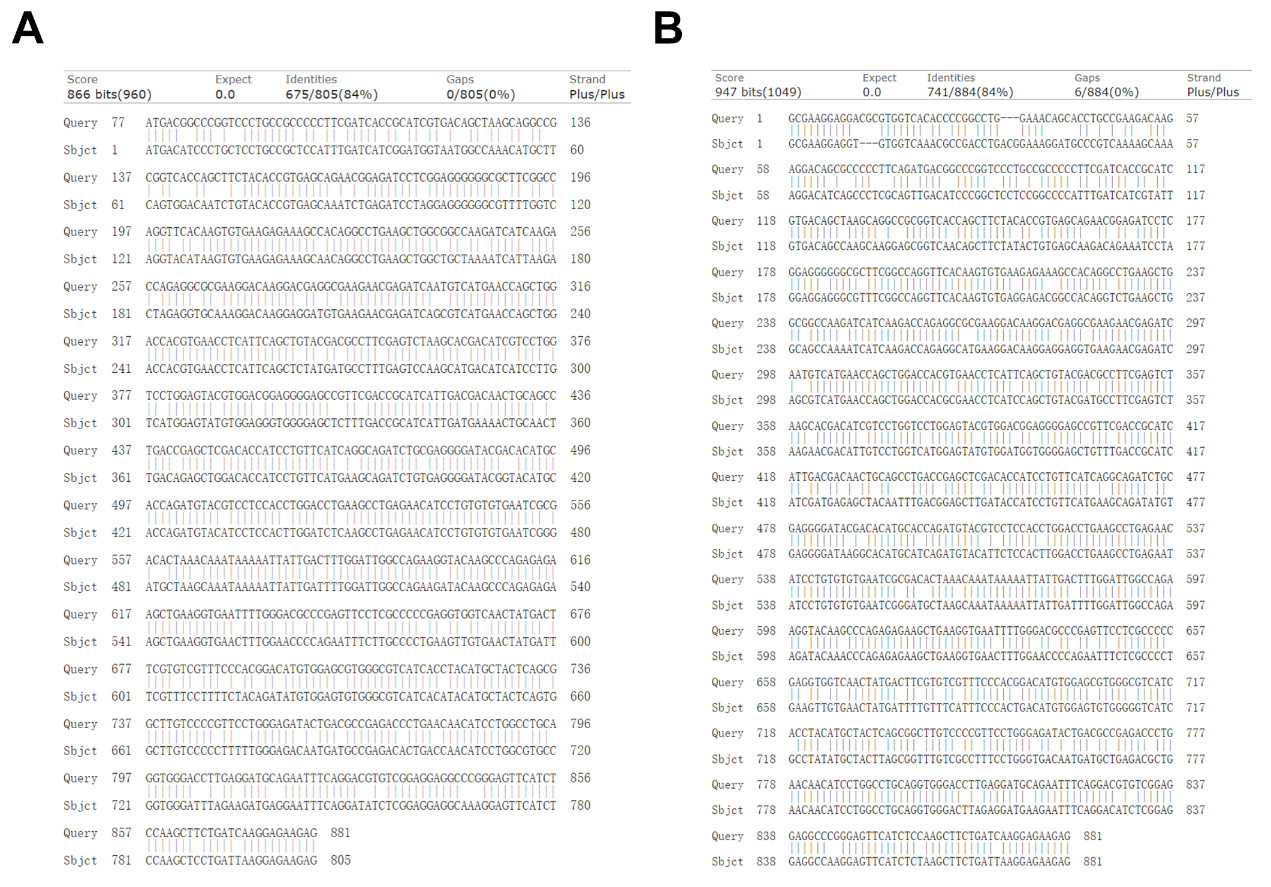


**Fig. S1** Conservation analysis of circMYLK4. (A) Sequence alignment of circMYLK4 and the circular RNA mmu-Mylk4_0004 generated from mouse MYLK4. (B) Sequence alignment of circMYLK4 and the human MYLK4-generated circular RNA hsa-MYLK4_0016.


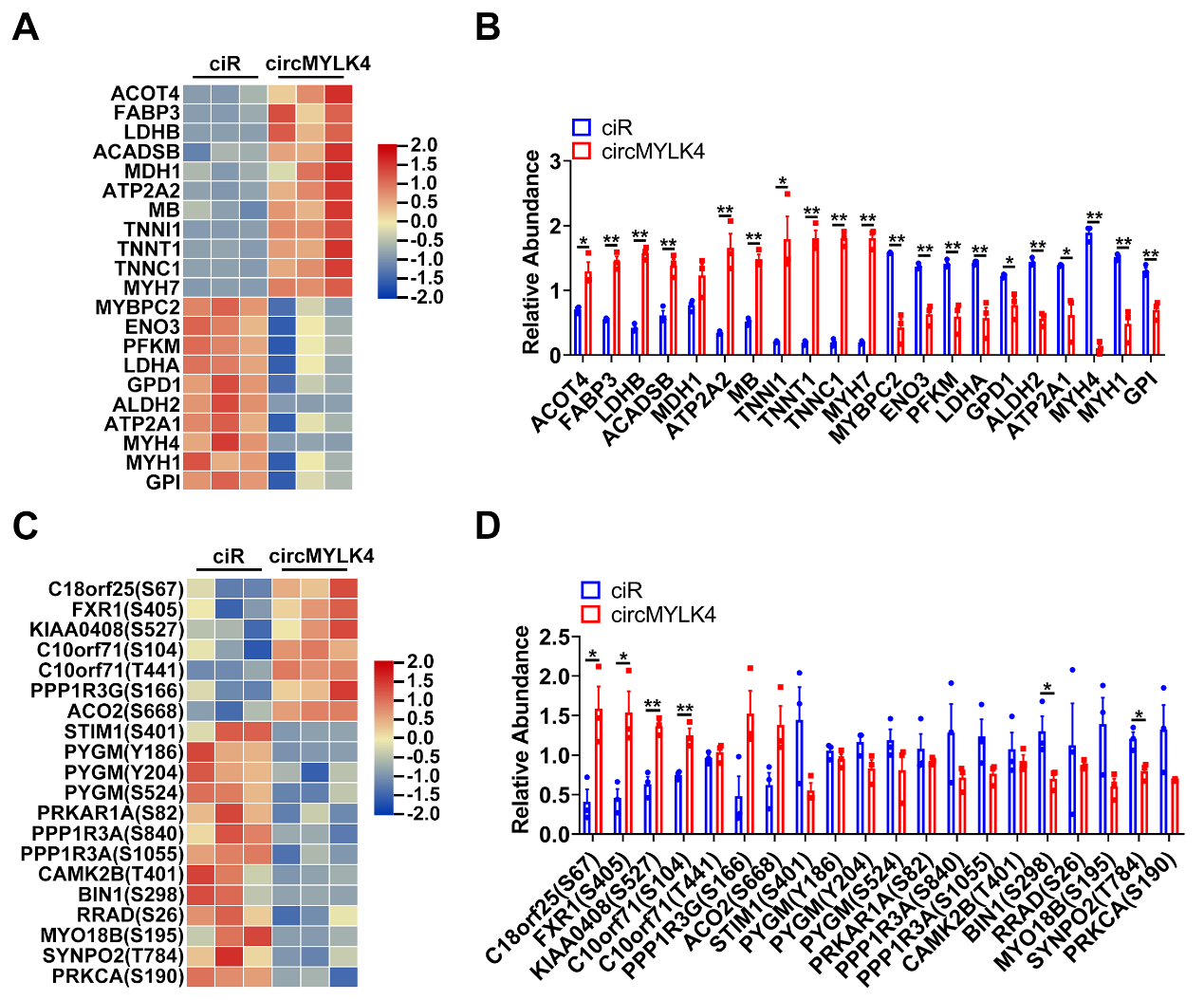


**Fig. S2** Validation of protein and phosphorylated protein profiles by PRM. (A) Twenty-one differentially expressed proteins randomly screened in proteomics. (B) Performing PRM validation on the selected 21 proteins. (C) Twenty differentially expressed phosphorylation sites randomly selected in phosphoproteomics screening. (D) Performing PRM validation on the selected 20 phosphorylation sites.


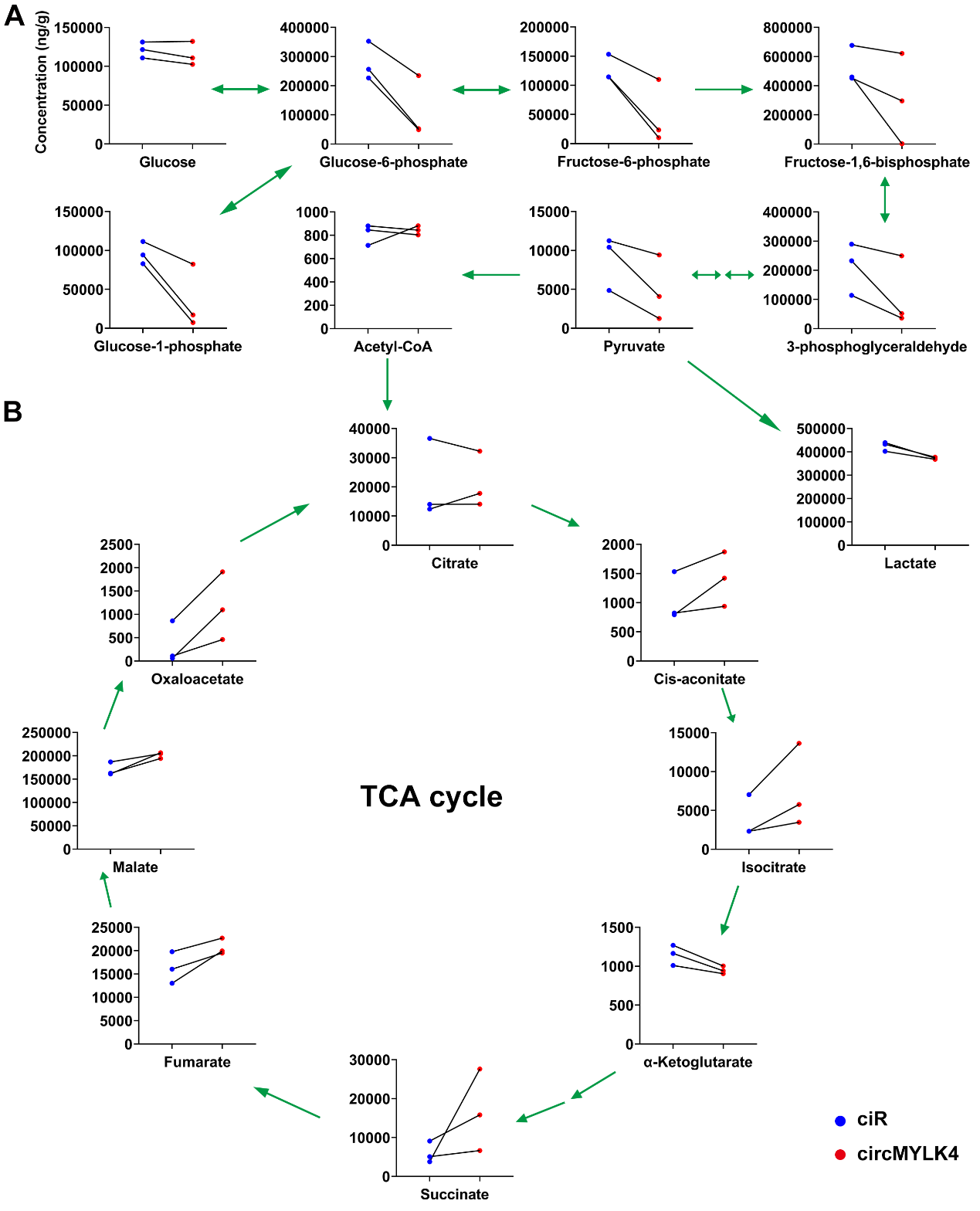


**Fig. S3** Metabolic analysis of skeletal muscle overexpressing circMYLK4. (A) Changes in the concentrations of key metabolites involved in glycolysis in skeletal muscle overexpressing circMYLK4. (B) Changes in the concentrations of key metabolites involved in the TCA cycle in skeletal muscle overexpressing circMYLK4.


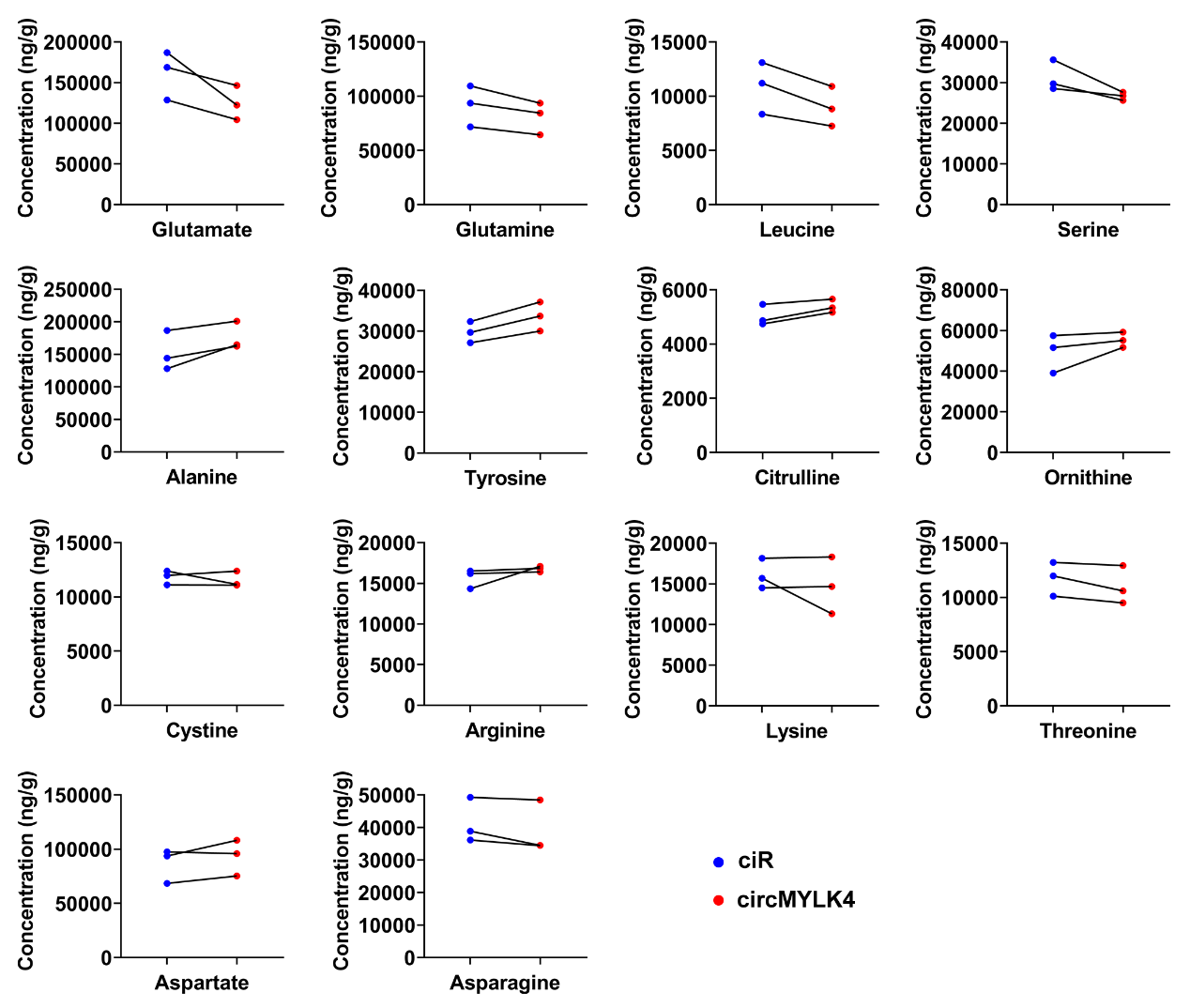


**Fig. S4** Changes in the concentrations of amino acids in skeletal muscle overexpressing circMYLK4.


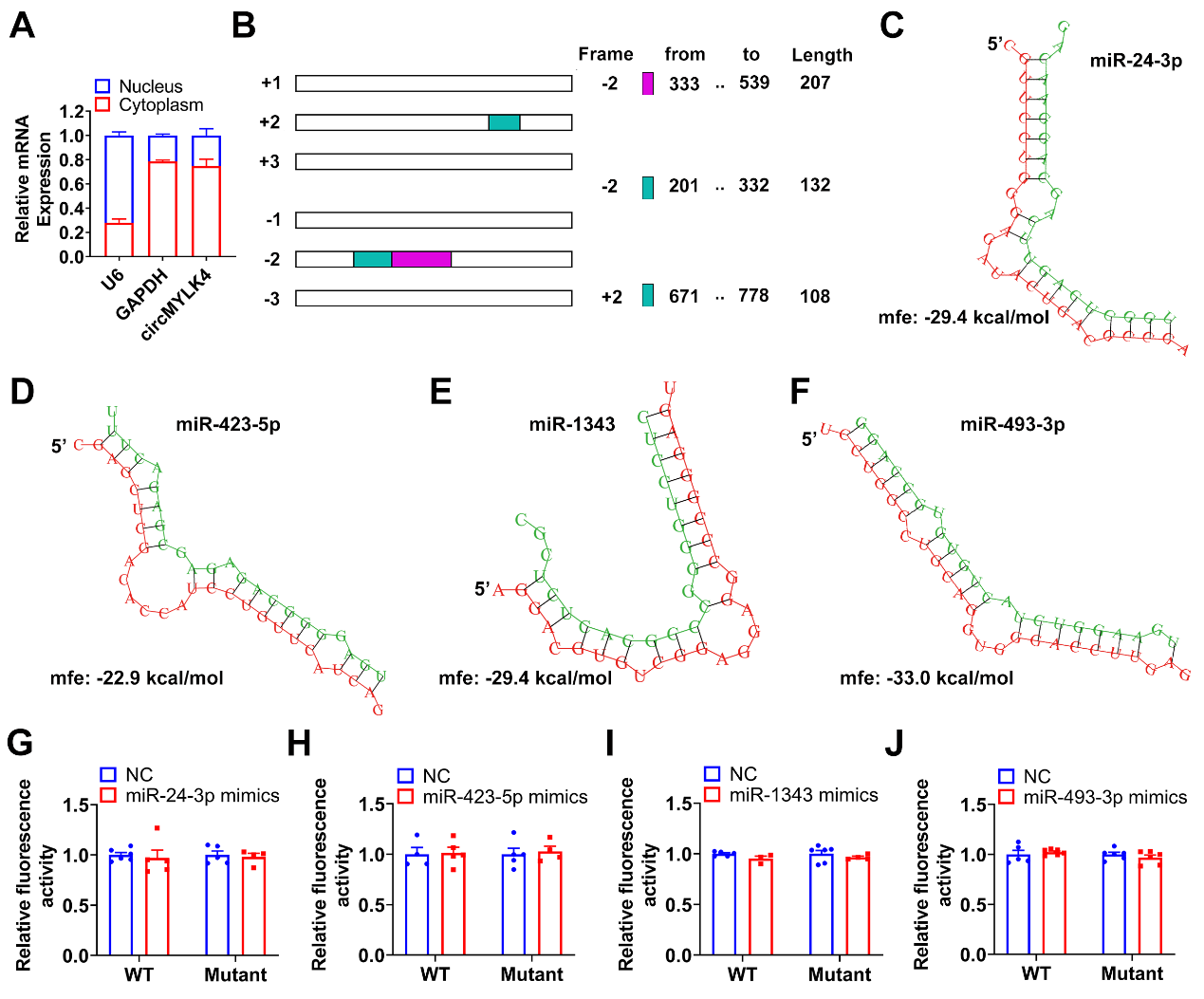


**Fig. S5** Mechanistic investigation of circMYLK4. (A) Nuclear-cytoplasmic fractionation to determine the subcellular localization of circMYLK4. (B) Prediction of the open reading frame of circMYLK4. (C-F) Prediction of the binding sites of circMYLK4 with miR-24-3p, miR-423-5p, miR-1343, and miR-493-3p. (G-J) Dual-luciferase assay to validate the binding of circMYLK4 with miR-24-3p, miR-423-5p, miR-1343, and miR-493-3p.


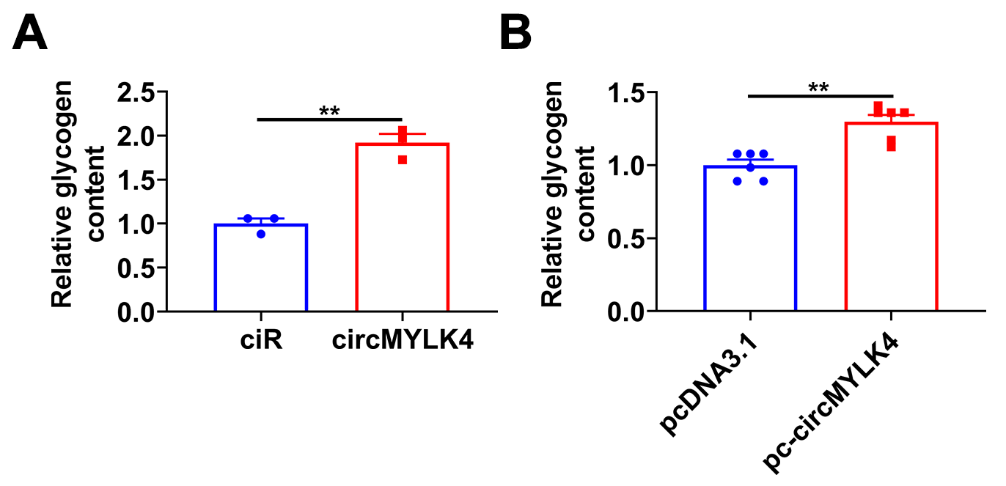


**Fig. S6** Measurement of glycogen content. (A) Measurement of relative glycogen content in skeletal muscle overexpressing circMYLK4. (B) Measurement of relative glycogen content in muscle satellite cells overexpressing circMYLK4. Data are mean ± SEM of three independent experiments (^*^*P* < 0.05, ^**^*P* < 0.01)
